# DESI-MSI-guided exploration of metabolic-phenotypic relationships reveals a correlation between PI 38:3 and proliferating cells in clear cell renal cell carcinoma via single-section co-registration of multimodal imaging

**DOI:** 10.1007/s00216-024-05339-0

**Published:** 2024-05-23

**Authors:** Greice M. Zickuhr, In Hwa Um, Alexander Laird, David J. Harrison, Alison L. Dickson

**Affiliations:** 1 School of Medicine, University of St Andrews, North Haugh, St Andrews, KY16 9TF UK; 2 Department of Urology, Western General Hospital, Crewe Road South, Edinburgh, EH4 2XU UK; 3 NuCana Plc, Lochside Way, Edinburgh, EH12 9DT UK

**Keywords:** Mass spectrometry imaging, DESI-MSI, Multiplex immunofluorescence, Image analysis, Multimodal imaging, AI

## Abstract

A workflow has been evaluated that utilizes a single tissue section to obtain spatially co-registered, molecular, and phenotypical information suitable for AI-enabled image analysis. Desorption electrospray ionization mass spectrometry imaging (DESI-MSI) was used to obtain molecular information followed by conventional histological staining and immunolabelling. The impact of varying DESI-MSI conditions (e.g., heated transfer line (HTL) temperature, scan rate, acquisition time) on the detection of small molecules and lipids as well as on tissue integrity crucial for integration into typical clinical pathology workflows was assessed in human kidney. Increasing the heated transfer line temperature from 150 to 450 °C resulted in a 1.8-fold enhancement in lipid signal at a scan rate of 10 scans/s, while preserving histological features. Moreover, increasing the acquisition speed to 30 scans/s yielded superior lipid signal when compared to 10 scans/s at 150 °C. Tissue morphology and protein epitopes remained intact allowing full histological assessment and further multiplex phenotyping by immunofluorescence (mIF) and immunohistochemistry (mIHC) of the same section. The successful integration of the workflow incorporating DESI-MSI, H&E, and immunolabelling on a single tissue section revealed an accumulation of ascorbic acid in regions of focal chronic inflammatory cell infiltrate within non-cancerous kidney tissue. Additionally, a strong positive correlation between PI 38:3 and proliferating cells was observed in clear cell renal cell carcinoma (ccRCC) showing the utility of this approach in uncovering molecular associations in disease pathology.

**Graphical Abstract:**

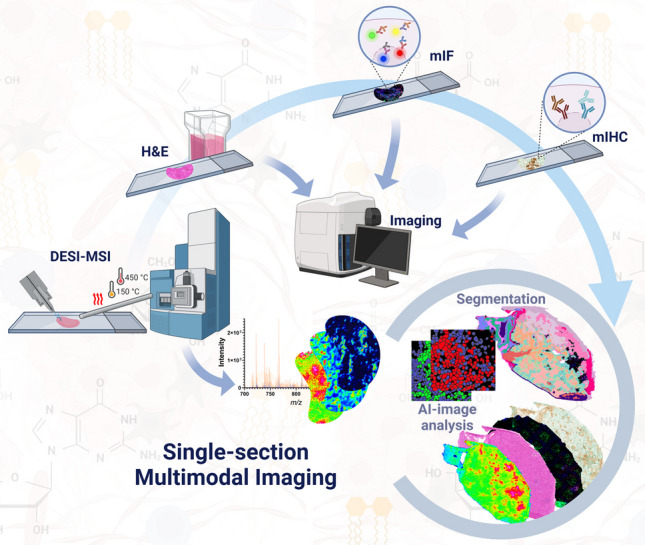

**Supplementary Information:**

The online version contains supplementary material available at 10.1007/s00216-024-05339-0.

## Introduction

Spatial metabolomic technologies are crucial for understanding biochemical processes involved in the pathophysiology of diseases by capturing the metabolic heterogeneity of cell types within their tissue environment as metabolites are direct markers of biochemical activities closely related to cell phenotypes [1, 2]. Mass spectrometry imaging (MSI)–based metabolomics methods can map the distribution of hundreds of these chemical species, and when coupled with other imaging modalities, MSI becomes a powerful technology. Conventional pathology techniques such as hematoxylin and eosin (H&E) stain, immunohistochemistry (IHC), and immunofluorescence (IF) provide complementary information on the spatial distribution of morphological features and specific protein markers [3].

DESI-MSI requires minimal sample preparation and is regarded as a “soft” ionization technology as its non-destructive nature on tissue has been demonstrated using a lab-built sprayer [4]. Since introduced, lab-custom and the commercial Prosolia design have been used to study different pathologies and shown to be compatible with different imaging modalities (e.g., H&E, IHC, IF, and imaging mass cytometry (IMC)) [5–9] allowing the obtention of phenotypical information from the same section submitted to MSI. Data co-registration from these different imaging modalities allows for comprehensively investigating complex biological systems and mechanisms by integration of ion images with histological features. These approaches, however, are limited to only one other imaging technique post-MSI and rely on adjacent sections for multiple imaging modalities, which requires registration and correlation of distinct non-identical sections [10–12]. Moreover, it has been shown that dimethylformamide and acetonitrile (DMF:ACN) is the optimal DESI solvent mixture for tissue histology preservation and that solvent mixtures containing methanol (MeOH) completely disrupt the tissue architecture [4].

MeOH mixtures, however, have been extensively used for DESI-MSI experiments due to their ability to provide enhanced signal intensity for lipids [13–16]. Additionally, MeOH is a well-established solvent for LC–MS experiments and is compatible with mass spectrometer systems. Nonetheless, compatibility issues with tissue histology deterred MeOH mixtures from usage in DESI-MSI experiments. More recently, though, studies have been utilizing high MeOH content solvent mixtures in DESI analysis followed by H&E [17–19]. Although the gross tissue structure appears to be preserved in these samples, no evaluation has been done on the cell level to identify potential tissue distortion and epitope degradation that would allow further immunolabelling on the same section. Furthermore, recently, a new commercial sprayer and heated transfer line (HTL) were introduced, but the impact of these recent technological improvements has not yet been assessed on tissue in a pathology or clinical setting [20]. Although DMF solvent mixtures yield spectra of relatively comparable quality, they favor low molecular mass compounds’ detection [4]. Moreover, due to its higher surface tension and boiling point compared to MeOH’s, DMF mixtures tend to produce larger spot sizes resulting in enlarged extraction areas and lower spatial image resolution, an effect that could be further intensified by the temperature functionality of the DESI XS system and HTL [4, 13, 21]. In this study case, we report a workflow that utilizes a single tissue section to obtain high molecular and phenotypical information, through multimodal imaging techniques, suitable for AI-enabled image analysis. We assessed the impact of varying DESI-MSI conditions using 98:2 (v/v) MeOH:H_2_0 as the desorption solvent on the quality of tissue for integration into typical clinical pathology workflows and imaging research. Using the multimodal imaging workflow, over 100 lipid species were spatially resolved in the tumor microenvironment of biopsies from three patients who had clear cell renal cell carcinoma (ccRCC), and image analysis was used to identify cell populations within tumor niches. To our knowledge, this approach is the first attempt to use DESI-MSI, histology, and immunolabelling on a single section to correlate different proteins’ expression and cell types with histological features and metabolic and lipidomic status.

## Materials and methods

### Materials and reagents

Polyvinylpyrrolidone (PVP) (MW 360 kDa), (hydroxypropyl) methyl cellulose (HPMC) (viscosity 40 − 60 cP and 2600–5600 cP), methanol, water, iso-pentane, and 2-propanol were of analytical grade or higher and purchased from Sigma-Aldrich (Steinheim, Germany). Hematoxylin (Mayers, pfm medical, UK), eosin Y 1% alcoholic and xylene (CellPath, UK), periodic-acid (TCS Biosciences Ltd, UK), and Schiff’s reagent (Merck, Germany). Details of the IF and IHC antibodies used can be found in the Supporting Information Table S1.

### Samples

Snap-frozen non-cancerous kidney tissue and clear cell renal cell carcinoma (ccRCC) biopsies were selected from partial nephrectomy undertaken as curative treatment of kidney cancer. Ethical approval was granted by Lothian Biorepository (SR1787 10/ES/0061).

### Tissue embedding and sectioning

Tissue was embedded in PVP (2.5%) modified HPMC (7.5%, 40–60 cP) which has been demonstrated previously by Dannhorn and colleagues to be compatible with tissue analysis [22]. Sectioning was performed on a dedicated (MSI use only) HM525 NX Cryostat (Epredia, Portsmouth, USA) to a thickness of 10 µm. Only mass spectrometry-compatible reagents were used in the cryostat to minimize the risk of source contamination. Serial sections were thaw-mounted onto Superfrost microscope slides (Thermo Scientific Waltham, MA), nitrogen-dried, vacuum packed, and stored at –80 °C. Prior to analysis, sections were left to equilibrate to room temperature under vacuum for 20 min.

### DESI-MSI experiments

Analysis was performed on a Xevo G2-XS Q-ToF equipped with a DESI-XS ion source and heated transfer line (Waters, Milford USA) operated at 20,000 resolving power in negative ionization mode between *m/z* 50–1200. A solvent mixture of 98% methanol and 2% water was delivered at 2 µL/min and nebulized with nitrogen at a backpressure of 1 bar. Spatial resolution was set at 20 × 20 µm. Imaging experiments were performed by setting the transfer line temperature to low 150 °C and high 450 °C, and scan speeds of 10, 20, and 30 scans/s. ccRCC samples were analyzed at 10 scans/s with the HTL set at 450 °C.

Data processing and visualization were performed in HDI® (Waters, Milford USA) for non-cancerous tissue and in SCiLS Lab 2024a (Bruker Daltonics, Germany) for ccRCC samples and normalized to TIC. Peak picking was performed with an *m/z* window of 0.02 Da and tentative compound assignments were made with high mass accuracy measurements (≤ 5 ppm mass error) using the Human Metabolome Database and Lipid Maps®. Tissue segmentation (UMAP) was performed using the Waters MicroApp MSI Segmentation, version 2.1.1 (Waters, Milford USA).

### Histochemical staining, multiplex immunofluorescence, and multiplex immunohistochemical labelling and imaging

Following DESI-MSI, tissues were stained by hematoxylin and eosin (H&E) and/or periodic acid–Schiff (PAS). An immunofluorescence panel containing NucBlue™ for nuclear identification and antibodies against p57 (glomerular podocytes), PFKFB3 (a marker of glycolytic activity), and HIF1α (a marker of hypoxia) were optimized separately and applied to sections following DESI-MSI and histochemical staining (Table S1). Fluorophores were stripped off the tissues and a mIHC panel of vimentin and pan-cytokeratin was applied. Additional optimized panels for CD45^+^ CD8^+^ mIF for confirmation of lymphocytes were applied to non-cancerous cases of immune infiltration and for Ki67^+^, CD45^+^, and CD3^+^ were applied to ccRCC cases. For detailed method information, see the Supporting Information. Brightfield images of H&E, PAS, and mIHC stains and fluorescence images of mIF were acquired using a Zeiss Axio Scan Z1 scanner. A uniform scanning profile was used for each fluorescence channel. QuPath [23] was used to visualize and export high-resolution brightfield images.

### AI-enabled image analysis

Brightfield and fluorescence images were analyzed using HALO® v3.6.4134.166 and HALO® AI v3.6.4134 (Indica Labs). For mIF image analysis, a customized nuclear segmentation classifier was built using the NucBlue™ channel as a guide to negate falsely segmented nuclei [24]. Fluorescence intensity thresholds were set individually for each channel using the nuclear marker as a guide.

## Results

**Fig. 1:**
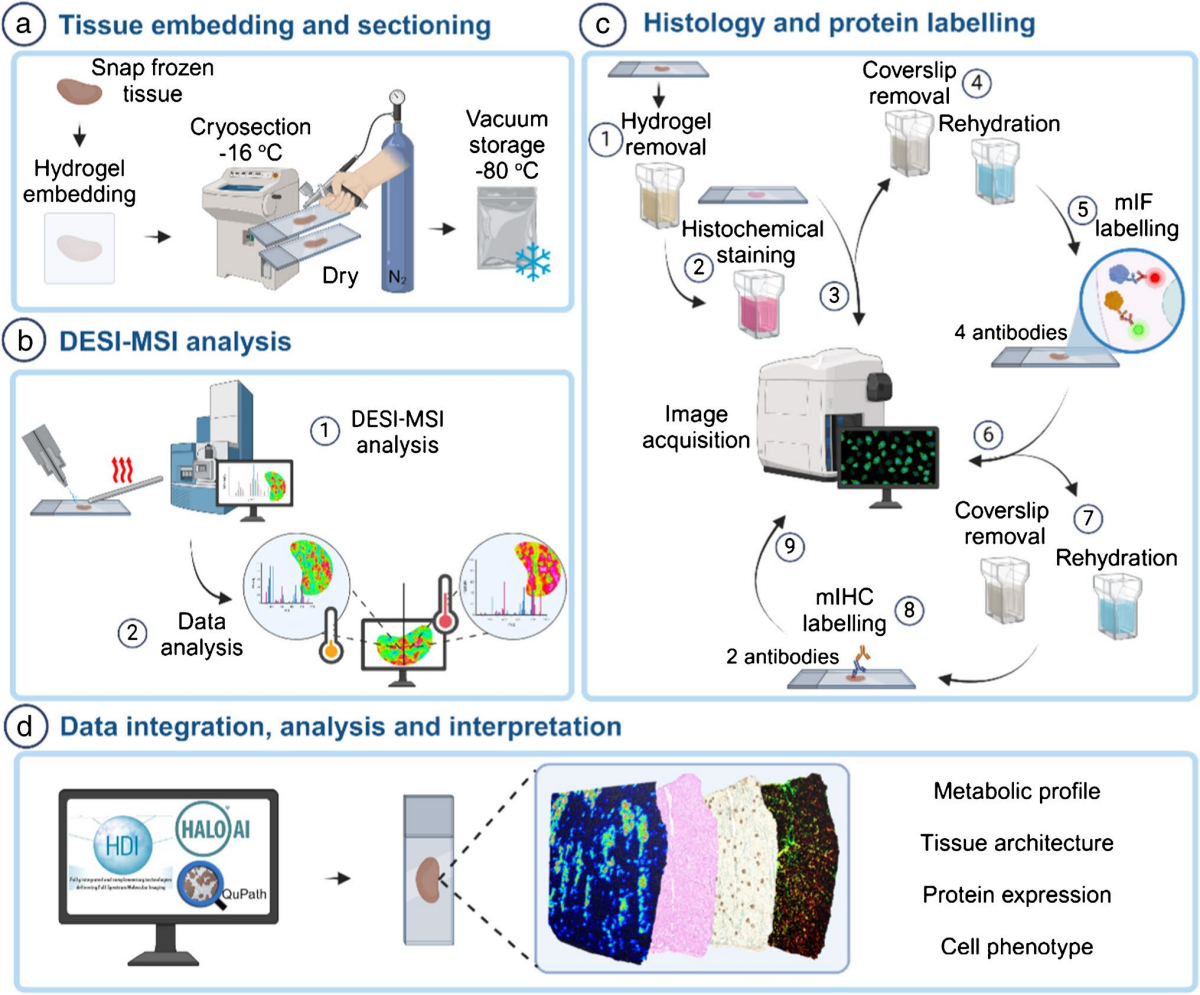
Schematic representation overview of the single-section multimodal imaging analysis pipeline. **a** Tissue section preparation for DESI-MSI; **b** tissue analysis by DESIXS MS with the re-designed Waters™ high-performance (HP) sprayer and heated transfer line (HTL); **c** post-DESI-MSI histochemical staining, multiplex immunofluorescence (mIF) and immunohistochemistry (mIHC) labelling steps and image acquisition; and **d** analysis, integration, and interpretation of data using HDI™, HALO® AI, and QuPath for spatial correlation of metabolic profile and cell phenotype

A reproducible workflow for metabolomics analysis by DESI-MSI followed by histology and protein labelling on the same tissue section was optimized and evaluated (Fig. 1). A summary of all tested conditions and outcomes is provided in Table 1.

**Table 1:** Summary of the conditions tested with the DESI XS source and their effect on signal intensity, histology, and immunophenotyping

DESI XS tested parameters		Signal intensity	Effect on H&E^b^	PAS^c^ staining	mIF^d^	mIHC^e^
HTL^a^ temperature (scan rate at 10 scans/s)	150 °C	Greater signal intensity for lower molecular mass compounds (*m/z* 50—350 Da)	Preserved all morphological features with some coagulation and fixation of proteins	Intact basement membranes of capillary loops and tubular epithelium highlighted by PAS	Preserved epitopes	Preserved epitopes with a positive effect from the fixation mechanism on stain intensity of pan-cytokeratin and vimentin
	450 °C	Enhanced signal of higher molecular mass compounds (*m/z* 600—1000 Da)	Preserved all morphological features with stronger coagulation, fixation, and fragmentation of proteins	Intact basement membranes of capillary loops and tubular epithelium highlighted by PAS	Preserved epitopes, higher background fluorescence due to coagulated proteins	Preserved epitopes with stain intensity comparable to MeOH controls
Scan rate with HTL at 450 °C	20 scans/s	Overall signal intensity decreased with the increase in the scan rate but did not compromise peak shape and resolution in any of the three conditions	Preserved all morphological features with stronger coagulation and fixation of proteins	-	-	-
	30 scans/s		Lower disturbance of tissue histology with less fragmentation of coagulated proteins	-	-	-

^a^*HTL* heated transfer line

^b^*H&E* hematoxylin and eosin

^c^*PAS* periodic acid–Schiff

^d^*mIF* multiplex immunofluorescence

^e^*mIHC* multiplex immunohistochemistry

### Effect of heated transfer line temperature and scan rate on compound classes detected by DESI-MSI

**Fig. 2:**
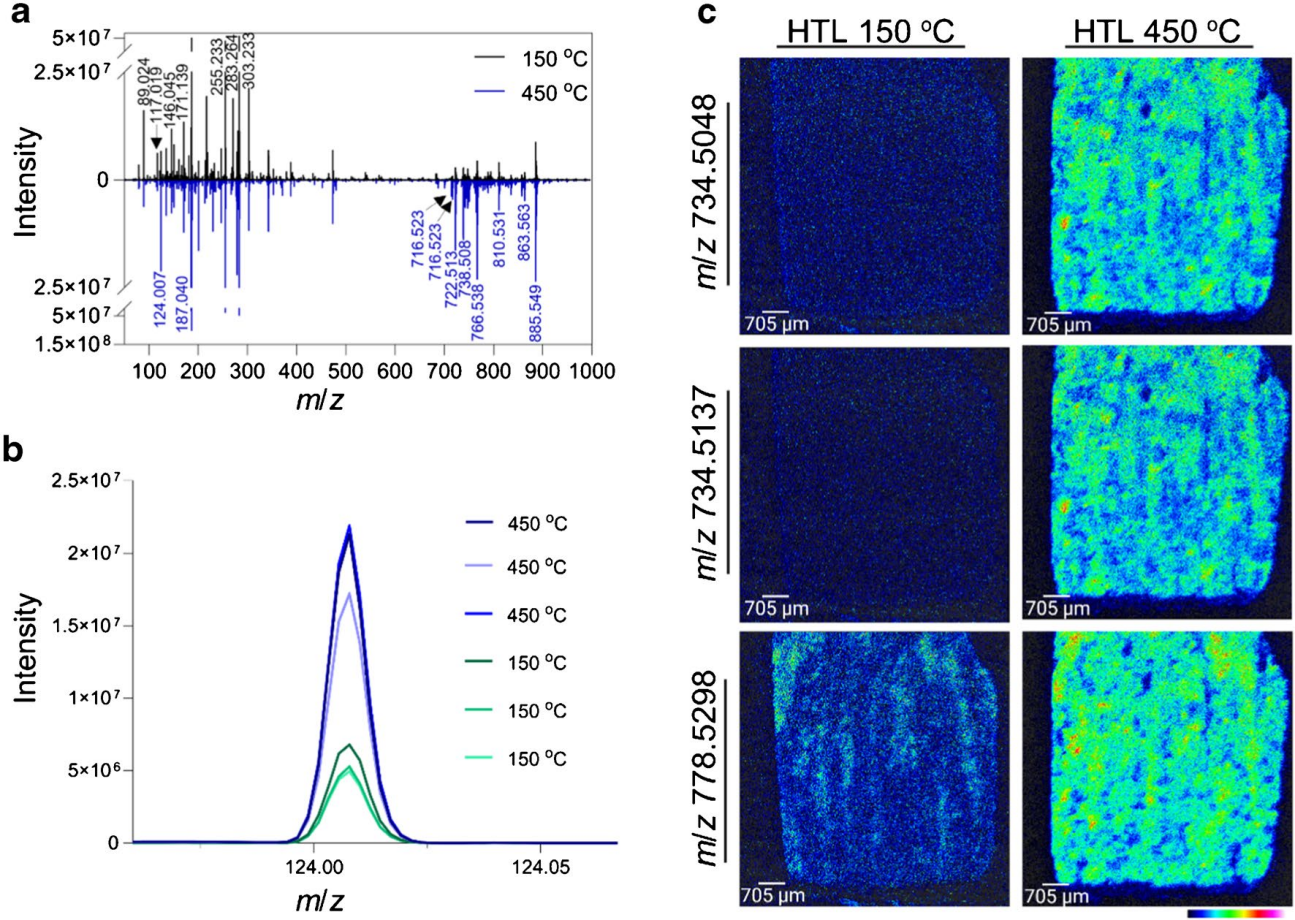
Effect of the DESI heated transfer line (HTL) temperature at 150 °C and 450 °C on different molecular classes of kidney tissue. Differences in **a** the negative ionization mode mass spectra of a kidney tissue analyzed with HTL at 150 °C (black) and 450 °C (blue); and **b** mass spectrum of *m/z* 124.0074 (taurine) showing higher intensity with HTL at 450 °C (*n* = 3). **c** DESI ion images showing increased sensitivity for *m/z* 734.5048, *m/z* 734.5137, and *m/z* 778.5298 with the HTL at 450 °C in comparison to 150 °C

To determine the effect of the heated transfer line (HTL) on the signal intensity of different molecular classes, kidney tissue sections were analyzed in triplicate with the HTL temperature set at 150 °C and 450 °C. The 1000 most intense *m/z* values from across all replicates generated a target list of 2455 features. A HTL temperature of 450 °C resulted in an increase of 1.8-fold in the overall signal intensity of species between *m/z* 600 and 1000 (e.g., lipids) (Fig. 2a). In this range, 55.0% (628 out of 1142) of the features had a boost in signal greater than 1.5-fold and 38.4% greater than twofold. Features presenting the highest increase in signal were distributed within the tissue, whilst the most significant drops in intensity were in background peaks (fold-change (FC) 450/150 < 0.09). Lipids were putatively identified based on their exact mass, and their distribution to kidney features was co-registered using H&E images (Table S4).

At the low *m/z* range (*m/z* 50–350), 488 peaks were detected, of which 52.3% had an average signal increase greater than 1.5-fold at 150 °C. Among these, we identified lactate (*m/z* 89.0246, FC 3.22), succinate (*m/z* 117.0193, FC 3.86), glutamate (*m/z* 146.0458, FC 2.25), and arachidonic acid (*m/z* 303.2330, FC 2.29). Intense *m/z* signals arise in this mass region due to the chemical properties of the microscope slides; this was also affected by the lowest transfer line temperature (e.g., *m/z* 255.2332 and 283.2645). Interestingly, the intensity of taurine (*m/z* 124.0074, Fig. 2b) was twofold higher at 450 °C whilst hypoxanthine (*m/z* 135.0311, FC 1.19) and ascorbic acid (*m/z* 175.0247, FC 0.78) were not highly influenced by the change in temperature. Figure 2c shows the effect of the sensitivity increase on the heatmaps of selected lipid ion species.

As a HTL temperature of 450 °C was shown to result in a higher signal for the lipid range, we decided to evaluate the effect on sensitivity and resolution of different scan speeds at this temperature. The acquisition speed was set to 10, 20, and 30 scans/s, with a fixed 20 × 20 µm pixel size, and resulted in average run times of 368, 216, and 143 min, respectively. A total of 6044 pixels approximately in the same area of the different scanned sections were extracted and raw data was examined. Although signal intensity reduced with the increase in scans/s, it did not significantly compromise the sensitivity (Fig. S1). In fact, at a speed rate of 30 scans/s with the HTL at 450 °C, spectra at the higher range (*m/z* 700–900) still resulted in higher signal intensity when compared to 150 °C at 10 scans/s (Fig. S2). Moreover, spectra show consistency in peak shape with low to no peak splitting between 10 to 30 scans/s, even for some lower-intensity species (Fig. S3).

### Effect of DESI sprayer and HTL temperature on tissue histology

To evaluate the effect of the DESI-XS high-performance (HP) sprayer in combination with the HTL on tissue histology, H&E and PAS staining were performed on previously DESI-MSI-scanned sections under different conditions. Data was acquired on three technical and three biological replicates scanned at 20 × 20 µm pixel size, at 10 scans/s, with HTL set to 150 °C or 450 °C. The effect of solvent was assessed by keeping paired control sections, for 10 min, in a solution of the solvent mixture. A tissue section was placed inside the enclosed DESI source during the run of its paired section to evaluate the atmospheric temperature and moisture effect. A third control was kept at room temperature for the period of the DESI-MS run. After DESI scanning, sections were H&E-stained and evaluated by an experienced kidney histopathologist (DJH) blinded to the conditions.

**Fig. 3:**
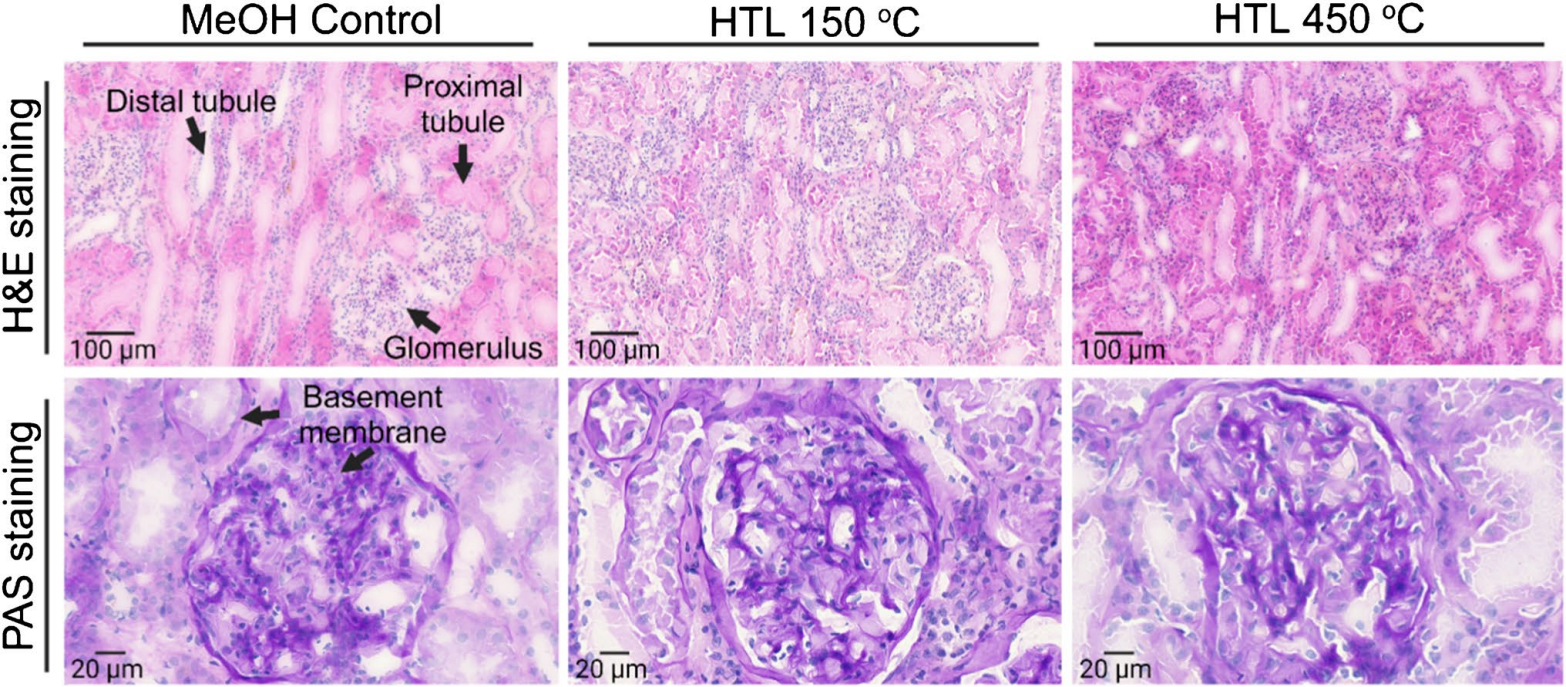
Histopathology assessment of kidney tissue sections post-DESI-MSI. Hematoxylin and eosin (H&E) stain of MeOH control and post-DESI analyzed tissue highlighting protein fixation as an effect of the interaction of solvent with tissue proteins and intensified by the higher temperature from the heated transfer line (HTL). Periodic acid*–*Schiff’s (PAS) staining on the same sections highlighting intact basement membranes post-DESI-MSI submitted to both HTL temperatures

Post-DESI-MSI analysis H&E and PAS-stained kidney sections (Fig. 3) retain features of the renal cortex and medulla of the human kidney including glomeruli, proximal and distal tubules, collecting ducts, blood vessels, medullary rays, and Bowman’s capsule.

Compared to the room temperature H&E controls, post-DESI sections and the MeOH controls show an intense eosin staining pattern with a more pronounced effect on the tissue submitted to analysis with the transfer line at 450 °C (Fig. 3 and Fig. S4). The combination of MeOH and high temperature results in coagulation and fixation of proteins on the tissue, mainly in the intratubular spaces, whilst in the H&E control these proteins have likely been washed out during the staining process. The individual effect of the atmospheric temperature inside the DESI source and the MeOH:H_2_O mixture is confirmed by the controls that show a similar, however less pronounced, coagulation pattern. Even though fixed, proteins are detaching from the tubules and vessels’ walls resulting in the loss of some fine morphology; the tissue features remain recognizable. To assess further the tissue integrity, PAS staining was used to highlight intact basement membranes of glomerular capillary loops and tubular epithelium across all DESI-MSI sampling conditions (Fig. 3 and Fig. S5). As a HTL of 450 °C was determined as the optimal condition for lipid analysis but most impactful on the morphology of the tissue, the impact from the combination of faster scan speeds at the higher transfer line temperature was evaluated. DESI-MSI carried out at 20 and 30 scans/s (Fig. S6) resulted in an identical protein coagulation and fixation pattern as seen previously at 10 scans/s. However, increasing the sprayer speed reduced protein fragmentation, suggesting lower disturbance on tissue histology in these conditions when compared to the slower scanning rate. This harshest effect at 10 scans/s in comparison to 30 scans/s agrees with the increased sensitivity observed in the MS results. The slower movement of the sprayer enhances the extraction of the sample components into the solvent increasing the intensity of the ionic signal, but also results in a stronger physical and chemical effect on the cellular and extracellular structures of the tissue. Although there is a loss in fine morphology observed in all sampled conditions, this loss has minimal effect on data interpretation.

### Epitope integrity confirmed by multiplex IF and IHC on the same tissue section post-DESI-MSI and H&E

**Fig. 4:**
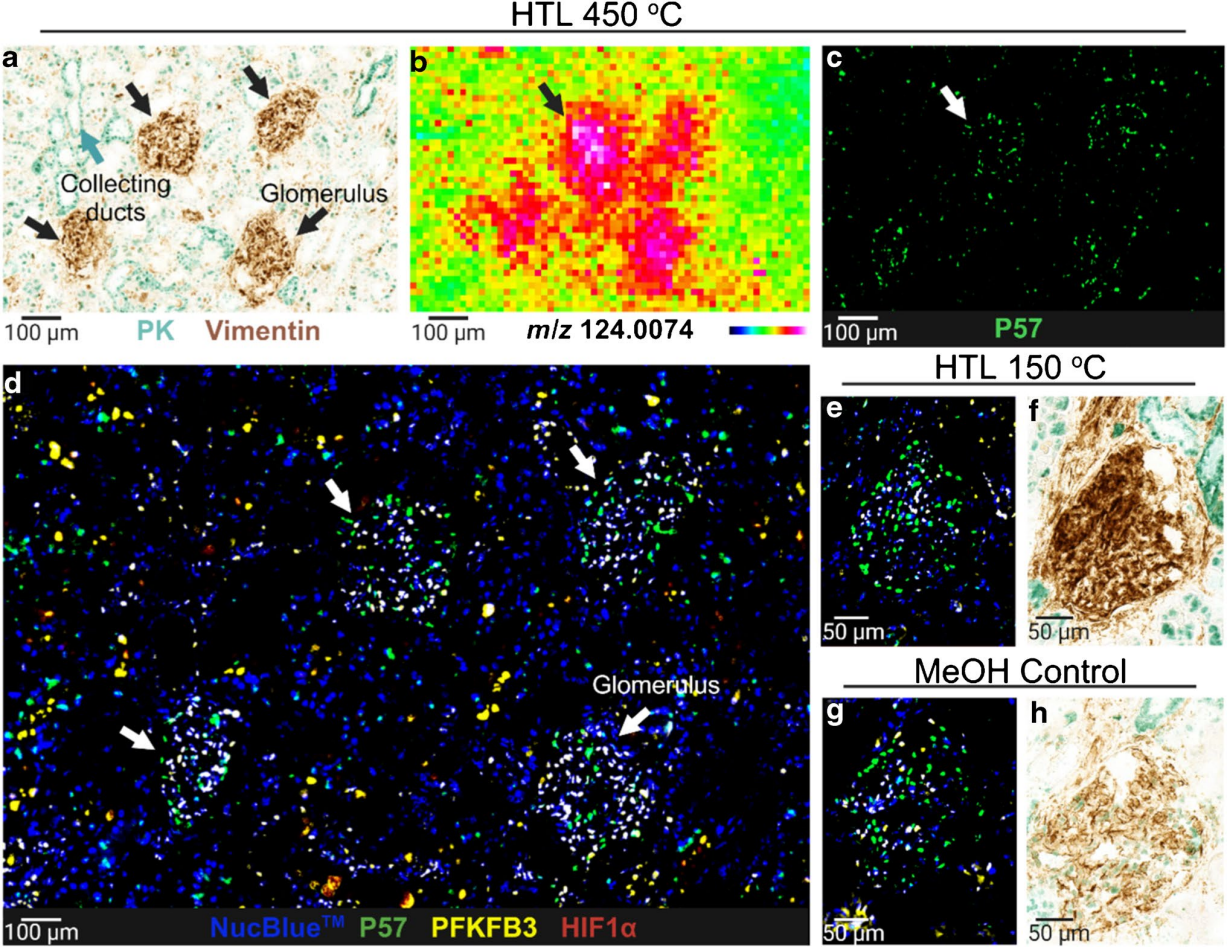
mIF and mIHC post-DESI-MSI. Protein epitopes and antibody binding for mIHC and mIF on kidney tissue remain intact post-DESI-MSI with the HTL at 150 °C and 450 °C. **a** mIHC image of collecting ducts (pan-cytokeratin^+^ (PK), green labelling) and glomeruli (vimentin^+^, brown labelling) post-DESI analysis with HTL at 450 °C, correlating to **b** higher taurine (*m/z* 124.0074) signal intensity and **c** podocytes (P57^+^) in glomeruli. **d** Composite of mIF-positive cells for P57 (podocytes, green labelling), PFKFB3 (glycolysis, yellow labelling), and HIF1α (hypoxia, red labelling) post-DESI with HTL 450 °C. **e**, **g** mIF and **f**, **h** mIHC image of the glomerulus of control and DESI analyzed section at 150 °C. NucBlue™ was used as the nuclear marker

After confirming tissue integrity through histology post-DESI imaging experiments, we next examined the impact of the desorption and different HTL temperatures on tissue phenotyping. For that, a four-plex immunofluorescence and a double IHC labelling panel were performed on post-DESI sections and MeOH controls after they were stained for H&E and/or PAS. As the scanning rate of 10 scans/s resulted in stronger morphological disturbance, it was chosen for this assessment. A total of twelve sections were successfully stained for all four IF and two IHC markers after DESI-MSI analysis. For mIF, each phenotype signal was individually assessed and co-registered with the nuclear marker that confirmed the protein expression to their corresponding cellular structures (Fig. 4). PFKFB3, HIF1α, and P57 showed strong nuclei staining. P57 was positive for cells showing weaker NucBlue™ staining, and PFKFB3 was predominantly expressed in glomeruli cells and some cells of collecting ducts, confirmed by co-expression with pan-cytokeratin (PK) in mIHC images. Strong vimentin (brown) expression was observed in glomeruli and vascular structures whilst pan-cytokeratin (green) labelled epithelial cells of the collecting ducts. Increased taurine distribution in the glomerulus, observed in DESI-MS images, was confirmed by co-registration with mIHC (vimentin) and mIF (P57).

Epitope dilutions were optimized for optimal signal intensities and kept consistent between cases and different DESI imaging conditions (Table S1). As previously confirmed by H&E on the same sections, 450 °C resulted in more protein fixation on the tissue leading to a higher background signal in the mIF images. This effect, however, was more pronounced in sections that were histochemical stained twice, by both H&E and PAS, with also lower pan-cytokeratin and vimentin staining intensity in IHC results (Fig. S7 and S8).

Immunohistochemical labelling was adequate for spatial data extraction after MSI, histology, and mIF for samples subjected to both 150 °C and 450 °C DESI analysis. Both vimentin and PK showed different staining intensities between controls and DESI-scanned sections. The fixation mechanism resultant from DESI at 150 °C appears to have a positive effect on IHC labelling by increasing the stain intensity. This effect was also seen in the sections that were PAS-stained (Fig. S8). Despite more severity of protein fixation and detachment at 450 °C, this did not negatively impact IHC staining as images from post-DESI-MSI and their MeOH control are comparable.

### Effect of DESI-MSI on image analysis by AI-enabled software

**Fig. 5:**
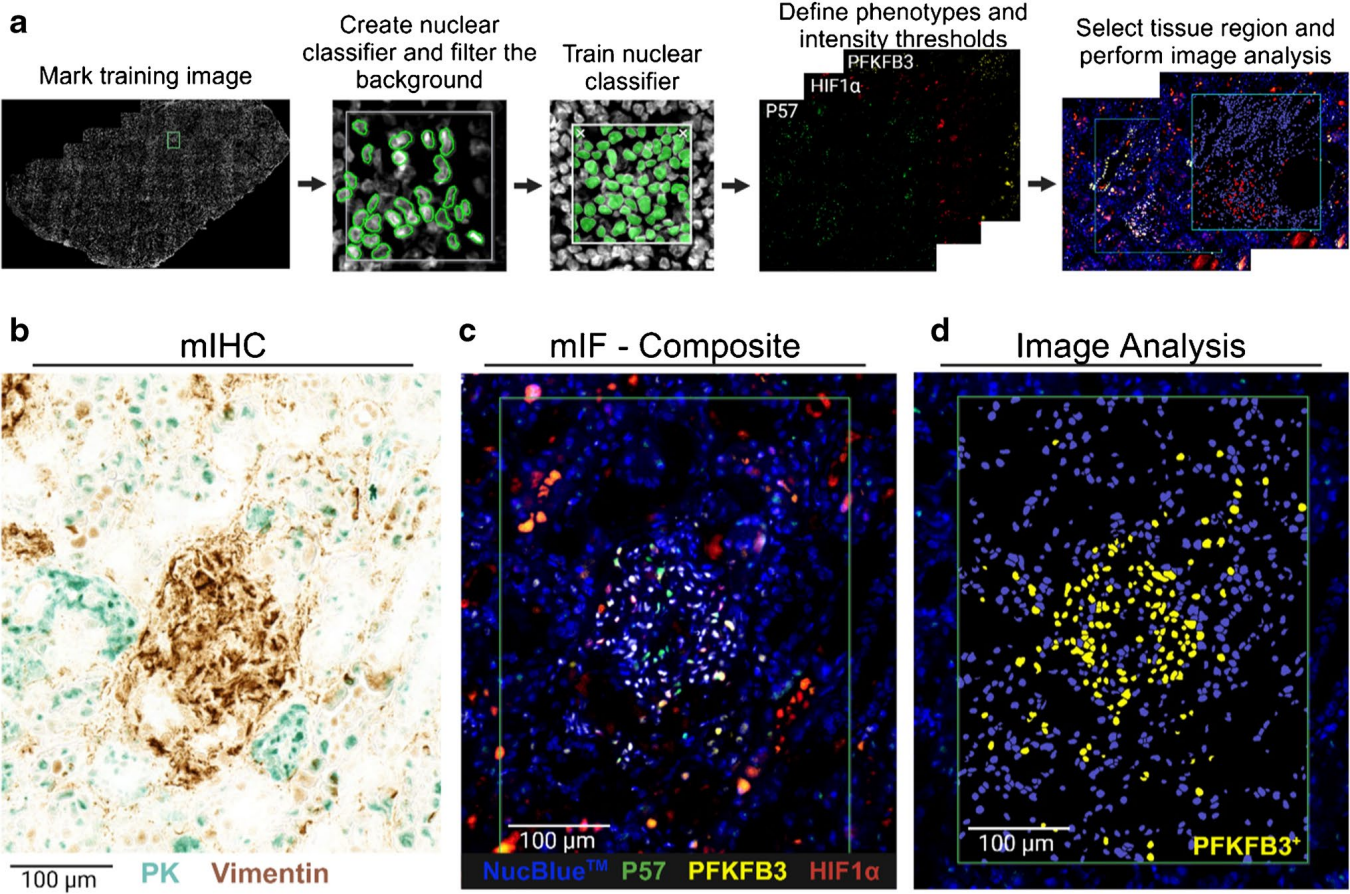
Image analysis. **a** AI-enabled (HALO AI®) image analysis workflow of mIF data. **b** mIHC labelling of epithelial cells of the collecting ducts with pan-cytokeratin (PK, cyan chromogen) and glomerulus with vimentin and same region. **c** mIF composite image of podocytes (P57^+^, green labelling), PFKFB3^+^ (yellow labelling), and HIF1α^+^ (red labelling) cells (NucBlue™—nuclear marker). **d** Nuclear segmentation and PFKFB3^+^ cells identified by AI-enabled image analysis. Tissue was analyzed by DESI-MSI at 10 scans/s with the HTL at 450 °C and H&E-stained prior to mIF and mIHC

Following the pathologist’s evaluation, AI-enabled image analysis was used to identify and segment positive cells in the glomeruli for the three mIF markers. Figure 5a illustrates the workflow for image analysis where firstly a nuclear segmentation classifier was built and trained. Subsequently, a threshold was set for each individual channel using nuclear staining (NucBlue™) as a reference to detect positive cells. As a result, image analysis accurately identified podocytes in the glomerulus and cells positive for PFKFB3 and HIF1α (Fig. 5d and S9) demonstrating segmentation and assignment can be performed on sections analyzed at the most extreme conditions of 450 °C.

### Application of the workflow

#### Single-section DESI-MSI, H&E, mIF, and mIHC indicate accumulation of ascorbic acid in areas of inflammation in non-malignant human kidney

**Fig. 6:**
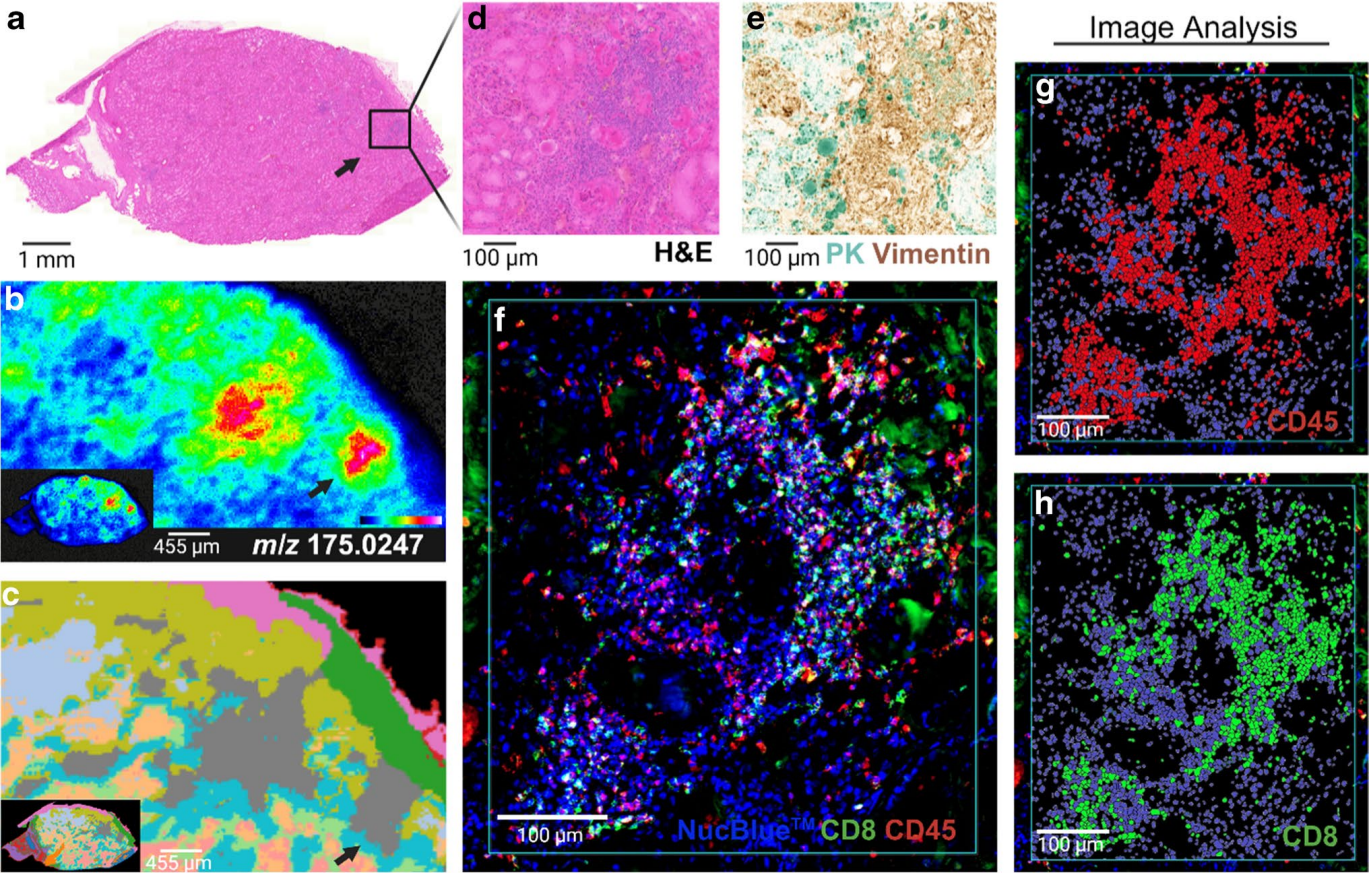
Differential metabolic fingerprints identified by DESI-MSI and UMAP segmentation confirmed immune infiltration by single-section multimodal imaging workflow. **a** Whole section H&E stain of a kidney tissue post-DESI scanning. **b** DESI-MS ion image of *m/z* 175.0247 (ascorbic acid) and **c** corresponding UMAP segmentation cluster of the MSI data. Detailed view of one of the areas with a higher concentration of ascorbic acid by **d** H&E showing fibrotic scars and immune cell infiltration; **e** mIHC labelling with pan-cytokeratin (PK, cyan labelling) and vimentin (brown labelling) expression indicating increased numbers of mesenchymal cells (fibroblasts) contributing to fibrosis and **f** composite image of mIF labelling of CD8^+^ (green) and CD45^+^ (red) cells. **g**, **h** Identified CD45^+^ and CD8^+^ cells through the application of HALO® AI image analysis to the mIF data. Section analyzed with HTL at 450 °C

DESI-MS image from one of the kidney sections previously analyzed showed a heterogeneous distribution of *m/z* 175.0247 (ascorbic acid) with some areas displaying higher intensity of the ion. When the dataset was subjected to a UMAP segmentation algorithm, 14 molecular clusters were identified. Of these, cluster 08 was found to spatially represent the area with increased ascorbic acid concentration. Re-evaluation of the H&E image revealed co-localization of a higher intensity of ascorbic acid in regions of arteriosclerosis and hyalinization of arterioles with focal chronic inflammatory cell infiltrate, consistent with chronic hypertension-related injury. To this section, we then applied a different mIF panel and by digital image analysis identified CD45^+^ and CD8^+^ cells in these regions to demonstrate a practical application of the evaluated workflow (Fig. 6). Co-expression with vimentin indicated increased number of mesenchymal cells (fibroblasts) contributing to the fibrosis identified by the pathologist [25, 26].

### Molecular mapping of lipids and image analysis indicates a possible correlation between PI 38:3 with Ki67^+^ cells in ccRCC

**Fig. 7:**
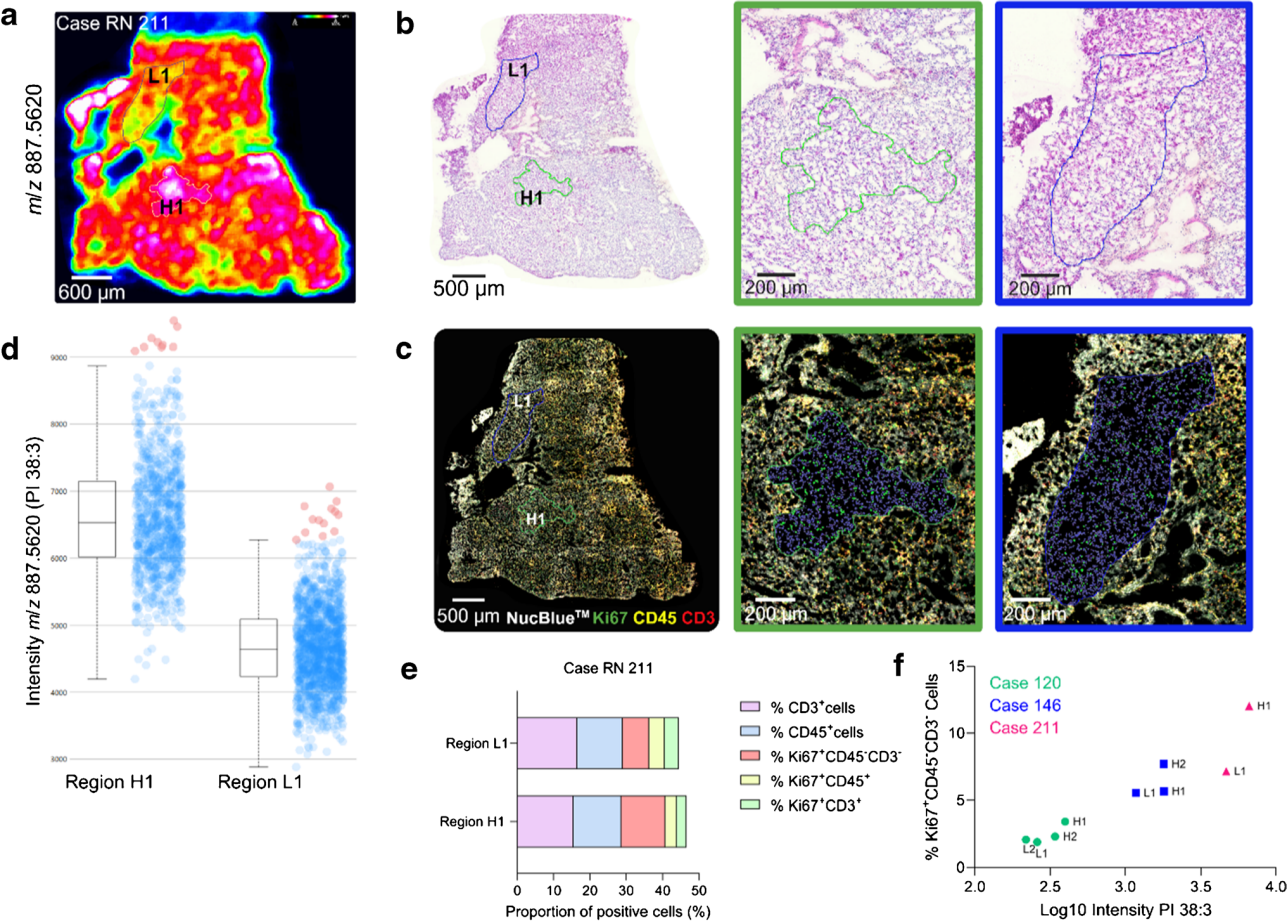
PI 38:3 intensity positively correlates with Ki67^+^ CD45^−^ CD3^−^ cells in ccRCC biopsies. **a** Ion image of *m/z* 887.5620 (PI 38:3) of case RN 211 and selected regions of interest (ROI) of high (H1) and low (L1) ion intensity. **b** H&E-stained section post-DESI-MSI highlighting ROI of high (H1) and low (L1) PI 38:3. **c** Composite image of mIF labelling, post-DESI and H&E staining, of Ki67^+^ (green), CD45^+^ (yellow), and CD3^+^ (red) cells and image analysis results of the ROI of high and low PI 38:3 highlighting in green Ki67^+^ CD45^−^ CD3^−^ cells. **d** *m/z* 887.5620 ion intensity of the analyzed ROI. **e** Percentage of positive cells for each analyzed phenotype amongst the total number of cells in the ROIs. **f** Logarithmic positive correlation of PI 38:3 average intensity with Ki67^+^ CD45^−^ CD3.^−^ cells in the analyzed ROI (two-tailed Spearman’s correlation analysis *r* = 0 0.93, *p* = 0.0007)

Next, to explore the potential of the workflow to study health disorders, we investigated the heterogeneity of various lipid classes in the tumor microenvironment (TME) of three human clear cell renal cell carcinoma (ccRCC) tissue samples. Across the *m/z* range of 300–1000 Da, we putatively identified an average of 118 ion species (data not shown). Notably, when analyzing the MSI data alongside the corresponding H&Es, we observed a heterogeneous distribution of *m/z* 887.5620, identified as PI 38:3 (lipid identity was confirmed by tandem mass spectrometry as phosphatidylinositol) (PI (18:0_20:3), DESI-MS/MS ion fragmentation is shown in Fig. S10), within case RN 211. Particularly intriguing were two regions exhibiting similar histology but significantly different ion intensities (Fig. 7a and b), a trend also evident in cases RN 120 and RN 146 (Fig. S11 and S12), although less pronounced.

To elucidate the heterogeneous distribution of PI 38:3 within regions of histological similarity, we investigated the association between high levels of PI 38:3 and regions of heightened cancer cell proliferation as reported in glioblastoma [27]. Thus, we investigated the relationship between PI 38:3 and Ki67^+^ labelling index (a marker of proliferation) in our ccRCC cases. As H&E images of cases RN 211 and RN 146 also showed immune cell infiltration, we performed mIF on the same sections to further characterize the immunophenotypes for CD45 and CD3, and Ki67.

Regions of interest (ROI) were selected based on the distribution of PI 38:3 ion intensity and histological similarity (Fig. 7 and Fig. S10 and S11). Co-registration of images derived from the same section, namely mIF and MSI, allowed image analysis (HALO® AI v3.6.4134) to identify Ki67^+^ CD45^−^ CD3^−^, Ki67^+^ CD45^+^ CD3^−^, and Ki67^+^ CD45^+^ CD3^+^ cells within the same ROI. The average intensity of PI 38:3 showed a log-linear correlation with the number of Ki67^+^ CD45^−^ CD3^−^ cells. A two-tailed Spearman correlation analysis (*r* = 0.93, *p* = 0.0007) revealed a significant positive correlation between these variables, though no such correlation was observed between PI 38:3 intensity and total cell density (*r* =  − 0.25, *p* = 0.52).

## Discussion

Integration of DESI-MSI into pathology workflows offers the potential to complement traditional histological techniques and enhance our understanding of disease processes. In this study, we explored the capacities of the re-designed DESI sprayer and HTL at different temperatures and developed and evaluated a workflow for its integration with multiple histology, mIF, mIHC, and digital image analysis for full histological assessment from a single tissue section of human kidney and cancer.

By increasing the HTL temperature to 450 °C, enhanced lipid spectra in the range of *m/z* 600–1000 were obtained along with preserved histological information. The high temperature of the HTL causes the temperature inside the enclosed DESI source to rise. As a result, all parts of the source system environment will increase in temperature which may result in a slight increase in the temperature of the solvent being delivered. Moreover, heat induces lipid structural flexibility, which can help in their desorption from tissue [28]. Both phenomena and aided desolvation of secondary droplets in the HTL may explain the increase in sensitivity for this class of molecules [29]. This, however, was not a global metabolite effect, as the signal of most small molecule species (*m/z* 50–350) reduced with increasing HTL temperature. While we observed a positive correlation between the sensitivity of certain small molecules, such as taurine, and an increase in the HTL temperature, we did not extensively investigate this class of molecules. However, it has been previously shown that several of these molecules can be thermally unstable [30]. Therefore, care should be taken when interpreting data from samples submitted to heated conditions as there is potential for an artefactual effect because of in-source fragmentation, ion competition, desorption degradation due to the high temperature of the HTL or on-tissue degradation, and transformation resulting in accumulation of certain molecules, as tissues sat in the DESI enclosed source under a heated atmosphere for several hours during analysis.

Long acquisition time is one of the limitations of MSI technologies. Patient-derived biopsies, however, are usually small specimens of tissue that generally require less analysis time. In addition, changing MSI acquisition parameters helps reduce data acquisition time. Analysts, nonetheless, must be aware that these changes may sacrifice sensitivity and spatial resolution. By using the novel DESI-XS sprayer in conjunction with the HTL and increasing scan speeds from 10 to 30 scans/s, we have obtained MSI results almost threefold faster with no significant loss of sensitivity and resolution. Furthermore, sacrificing sensitivity for faster runs when using the HTL still resulted in a better lipid signal than at a slow acquisition rate at lower temperature. This, for small areas of tissue, such as patient biopsies, could offer runs of just a few minutes maintaining high-quality data.

Although some studies have reported the integration of chemical or immune labelling of the same tissue following DESI-MSI, no studies have assessed the impact of the re-designed DESI XS sprayer, the use of high-content MeOH, or multiple histopathology techniques on the quality of the tissue to enable complete co-registration from multiple imaging modalities on a single-section [4, 9]. In our study, immunofluorescence labelling and histochemical staining revealed temperature and solvent effects on stain intensities, which did not affect histology interpretation and phenotyping. Through H&E and PAS, standard stains used in kidney biopsy, protein coagulation was highlighted in the kidney tubular structures, with an intensified effect with the HTL at 450 °C. In contrast to the first study that demonstrated that MeOH significantly damages the tissue histology [4], here, by using the DESI-XS source complete morphological information was preserved using 98:2 (v/v) MeOH:H_2_0, even with the added effect of the HTL at 450 °C. Though some protein fragmentation from the tubules was observed in all sampled conditions, the interpretation of results will hardly be affected due to the ~ 100-fold difference in resolution between the DESI and H&E generated images (20 × 20 µm and 0.2 µm pixel size, respectively).

For mIF and mIHC, although intensity differences were seen as a result of the DESI analysis, pre-analytical steps must also be considered as a cause. Though we have optimized the cryosection step to obtain the same thickness sections, variability is still present and is operator-dependent. This is an important step in MSI and histochemistry and must be carefully considered as thicker sections can result in more severe tissue shattering. Furthermore, tissue thickness is known to positively affect the fluorescence intensity [31], and with the sprayer and high temperature, higher intratubular protein coagulation may result in an increase of background signal. Despite the color inconsistency resultant of different DESI-MSI conditions and pre-analytical variables, AI segmentation and phenotyping was successful in both mIF panels we applied.

An analysis of fresh-frozen tissue by MSI typically involves using sections that are 10–20 µm thick [7, 14, 32]. However, when using serial sections for phenotyping, there is a significant risk of displacing regions of interest (ROI), particularly when considering small cell populations [33]. By using the same section from MSI, it is possible to achieve a deeper level of multimodality while ensuring correct co-registration. In this respect, we presented a workflow incorporating mass spectrometry imaging (MSI) for co-registration of metabolic changes with molecular markers aided by machine learning applied to a single section of both non-malignant human kidney and clear cell renal cell carcinoma, demonstrating the potential for DESI-MSI to secure its position as a critical tool in next-generation pathology, uniquely being able to describe function as well as structure and cellular composition.

The application of our workflow to ccRCC samples revealed a positive correlation between PI 38:3 and Ki67^+^ CD45^−^ CD3^−^ cells, similar to findings from a previous study in glioblastoma [27]. In line with a study in breast cancer, which identified elevated levels of PI 38:3 in regions of invasive and actively migrating cancer cell populations [34], our results suggest that PI 38:3 may play a crucial role in cell proliferation in ccRCC. Considering the dynamics within distinct tumor microenvironments, including niche-specific variations, we plan to expand our analysis to a larger cohort of samples to investigate the correlation between this lipid and cellular proliferation within tumor cells and cancer aggressiveness. Given the modest expression of Ki67 in all the analyzed ROI, the use of consecutive sections for mIF could have veiled the differences we observed, but by using the same section we could directly correlate the ion intensity to the labelling index for the same region.

This approach highlights the potential of DESI-MSI for studying dynamic crosstalk in the tumor microenvironment, biomarker discovery prediction of disease progression, and clinical outcomes [35].

### Supplementary Information

Below is the link to the electronic supplementary material.

Supplementary file1 (DOCX 22080 KB)

Supplementary file2 (XLSX 271 KB)
