# Supplementary material for: DESI-MSI-guided exploration of metabolic-phenotypic relationships reveals a correlation between PI 38:3 and proliferating cells in clear cell renal cell carcinoma via single-section co-registration of multimodal imaging
Source: Anal Bioanal Chem. 2024 May 23;416(18):4015–28. doi: 10.1007/s00216-024-05339-0 (PMC11249708; doi:10.1007/s00216-024-05339-0)
Supplement: Supplementary file 1 — Supplementary file1 (DOCX 22080 KB) [file 216_2024_5339_MOESM1_ESM.docx]

Supplementary Information

**DESI-MSI-guided Exploration of Metabolic-Phenotypic Relationships Reveals a Correlation Between PI 38:3 and Proliferating Cells in Clear Cell Renal Cell Carcinoma via Single-Section co-registration of Multimodal Imaging**

Greice M Zickuhr^1^, In Hwa Um^1^, Alexander Laird^2^, David J Harrison^1,3^ and Alison L Dickson^1,3^*

^1^School of Medicine, University of St Andrews, North Haugh, St Andrews, KY16 9TF

^2^Department of Urology, Western General Hospital, Crewe Road South, Edinburgh EH4 2XU, UK

^3^NuCana plc, Lochside Way, Edinburgh EH12 9DT, UK

* Corresponding Author ([ald24@st-andrews.ac.uk](mailto:ald24@st-andrews.ac.uk))

**Supplementary Methods**

Table S1. Antibody characteristics and dilutions

| **Methodology** | **Antibody** | **Company** | **Product code** | **Dilution** |
| --- | --- | --- | --- | --- |
| mIHC | Vimentin | Cell signalling tech | 5741 | 1:50 |
|  | Pan-cytokeratin | Dako | Z0622 | 1:50 |
| mIF | P57 | Santacruz | sc56341 | 1:100 |
|  | HIF1α | Abcam | ab51608 | 1:600 |
|  | PFKFB3 | Abcam | ab181861 | 1:200 |
|  | CD45 | Abcam | ab40763 | 1:500 |
|  | CD8 | Agilent | M710301-2 | 1:400 |
|  | CD3 | Agilent | A0452 | 1:500 |
|  | Ki67 (clone MIB1) | Agilent | M724001-2 | 1:200 |

**Method S1** Single-section histochemical staining, multiplex immunofluorescence and multiplex immunohistochemical labelling protocol.

**H&E**

After each DESI experiment, tissues were kept in 10% Tween® 20 detergent (TBST) buffer for 5 min to remove hydrogel, immersed in haematoxylin for 3 min, then washed in three consecutive water baths and immersed in TBST buffer for 1 min. Slides were then immersed in eosin for 10 sec, followed by consecutive washes in ethanol at 50, 80 and 100% baths. For dehydration, slides were immersed for 5 min in three different 100% xylene baths. Samples were then fixed with DPX glue and a coverslip. Brightfield images were acquired after samples were dried.

**PAS**

After H&E stained, selected sections were immersed in xylene until coverslip and all DPX glue was removed. Tissues followed a rehydration step of 2 min in 100, 80 and 50% ethanol and 2 min in distilled water. Slides were kept flat; sections were covered with 1% periodic acid for 5 min and then washed in tap and distilled water prior to being covered with Schiff’s reagent (1:4) for 10 min. Then sections were kept under warm running tap water for 5 min and counterstained with haematoxylin for 3 min following the same sequence above mentioned for H&E staining (skipping the eosin step) and fixed with a coverslip. Brightfield images were acquired after samples were dried.

**mIF**

Following H&E or PAS sections were uncovered in xylene solution overnight and kept in TBST prior to mIF staining.

The sections were transferred to the Leica Bond RX autostainer and automated multiplex immunofluorescence (mIF) was performed. The sections were treated with BOND epitope retrieval 1 (ER1) buffer (Leica, AR9961) for 20 min at 100 ^o^C to retrieve epitopes. Endogenous peroxidase activity and non-specific background stain were blocked by peroxide block (Leica, DS9800) and serum-free protein blocking buffer (Agilent, #X090930-2, Santa Clara, CA, USA), respectively. The first primary antibody, p57 (Santacruz, sc56341, 1:100), was incubated for 40 min, followed by post-primary and polymer (Leica, AR9961) for 20 min and 30 min, respectively. P57 was visualized by TSA fluorescein (Akoya Bioscience, #NEL741001KT, 1:200). Then, the sections were treated with ER1 buffer for 20 min at 95 ^o^C to remove redundant secondary and primary antibodies. The secondary primary antibody, HIF1α (Abcam, ab51608, 1:600) was incubated for 40 min, followed by polymer (Leica, DS9800) for 30 min. HIF1α was visualised by TSA Cyanine 5 (Akoya Bioscience, #NEL745001KT, 1:200). The sections were treated with ER1 buffer for 20 min at 95 ^o^C to remove redundant secondary and primary antibodies. The third primary antibody, PFKFB3 (Abcam ab181861, 1:100) was incubated for 40 min, followed by polymer (Leica, DS9800) for 30 min. HIF1α was visualised by TSA Cyanine 3 (Akoya Bioscience, #NEL744001KT, 1:200). Upon completion of mIF assays, sections were counterstained and mounted with ProLong™ glass antifade mountand with NucBlue (ThermoFisher, P36985Fluorescent images were acquired prior to mIHC.

**mIHC**

Post-mIF the sections were immersed in 0.1% TBST buffer at 40°C oven until the coverslips were removed. Sections were treated with BOND epitope retrieval 1 (ER1) buffer (Leica, AR9961) for 20 min at 95 ^o^C to remove redundant secondary and primary antibodies. Endogenous peroxidase activity and non-specific background stain were blocked by peroxide block (Leica, DS9800) and serum-free protein blocking buffer (Agilent, #X090930-2, Santa Clara, CA, USA), respectively. The fourth primary antibody, Vimentin (Cell signalling, 5741, 1:50), was incubated for 1 hour, followed by polymer (Leica, DS9800) for 30 min and was visualised by mixed DAB chromogen (Leica, DS9800) for 10 min. Then the redundant secondary and primary antibodies were stripped off with BOND epitope retrieval 1 (ER1) buffer (Leica, AR9961) for 20 min at 95 ^o^C. Endogenous peroxidase activity and non-specific background stain were blocked by peroxide block (Leica, DS9800) and serum-free protein blocking buffer (Agilent, #X090930-2, Santa Clara, CA, USA), respectively. The fifth primary antibody, pan Cytokeratin (Agilent, z0622, 1:50) was incubated for 1 hour, followed by polymer (Leica, DS9800) for 20 min and was visualised by Green chromogen (Leica, DC9913) for 10 min. Then the sections were counterstained with Haematoxylin (Leica, DS9800) for 5 min and mounted with prolonged gold anti-fade medium (ThermoFisher, #P36930). Brightfield images were acquired.

**Supplementary Results**


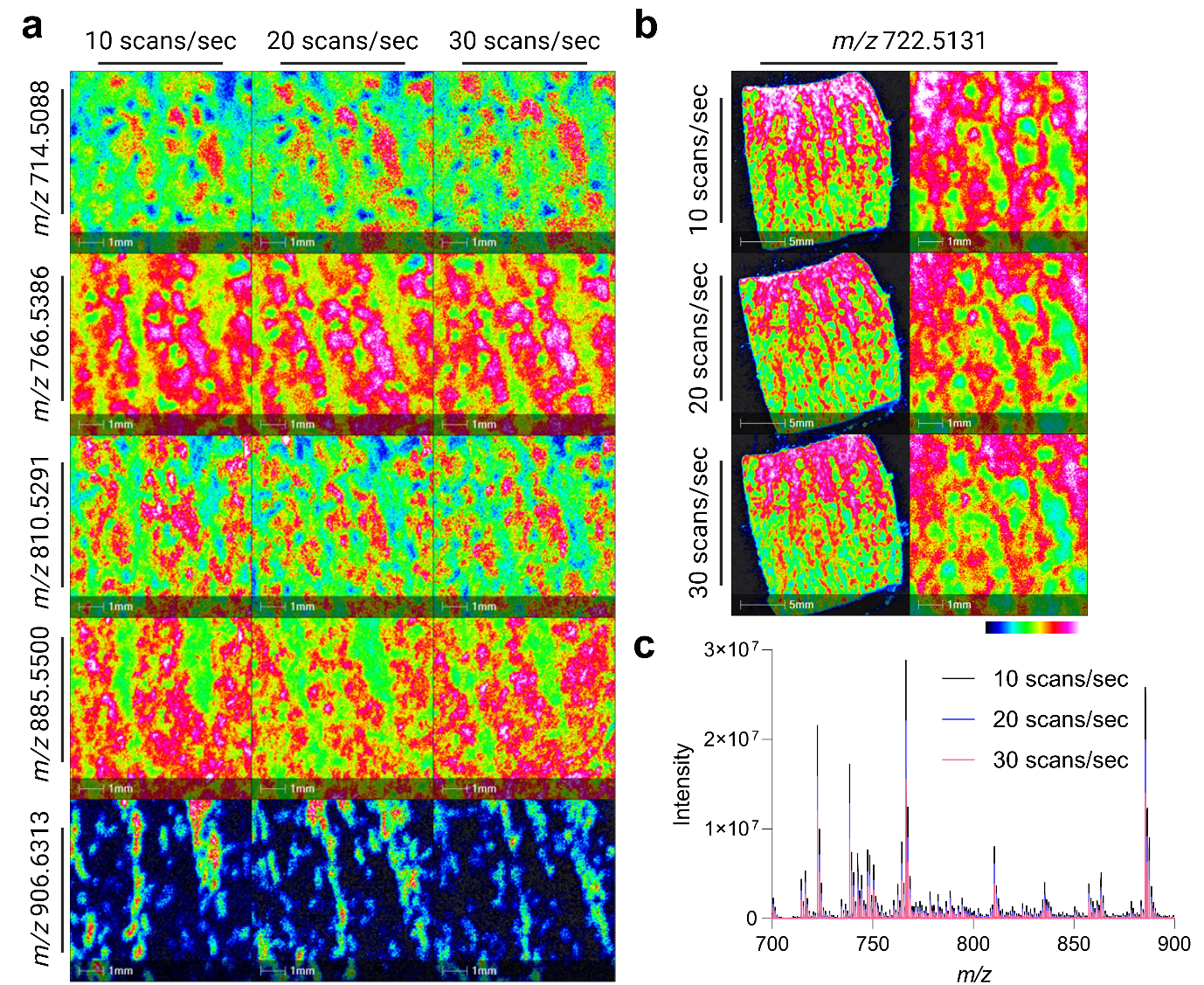


**Supplementary Fig. S1** **(a)** Ion images of lipids analysed by DESI-MSI with the HTL at 450 ^o^C at different scan speeds, m/z 714.5088 (PE 34:2), m/z 766.5386 (PE 38:4), m/z 810.5291 (PS 38:4), m/z 885.5500 (PI 38:4) and m/z 906.6313 (SHexCer 42:1;O3). **(b)** Whole human kidney tissue section and zoomed-in region ion images of m/z 722.5131 (PE O-36:5) analysed by DESI-MSI with the HTL at 450 ^o^C at different scan rates. **(c)** DESI-MSI spectra of kidney tissue sections in the range m/z 700-900 showing a decrease in sensitivity when increasing the acquisition speed when analysed with the HTL at 450 ^o^C


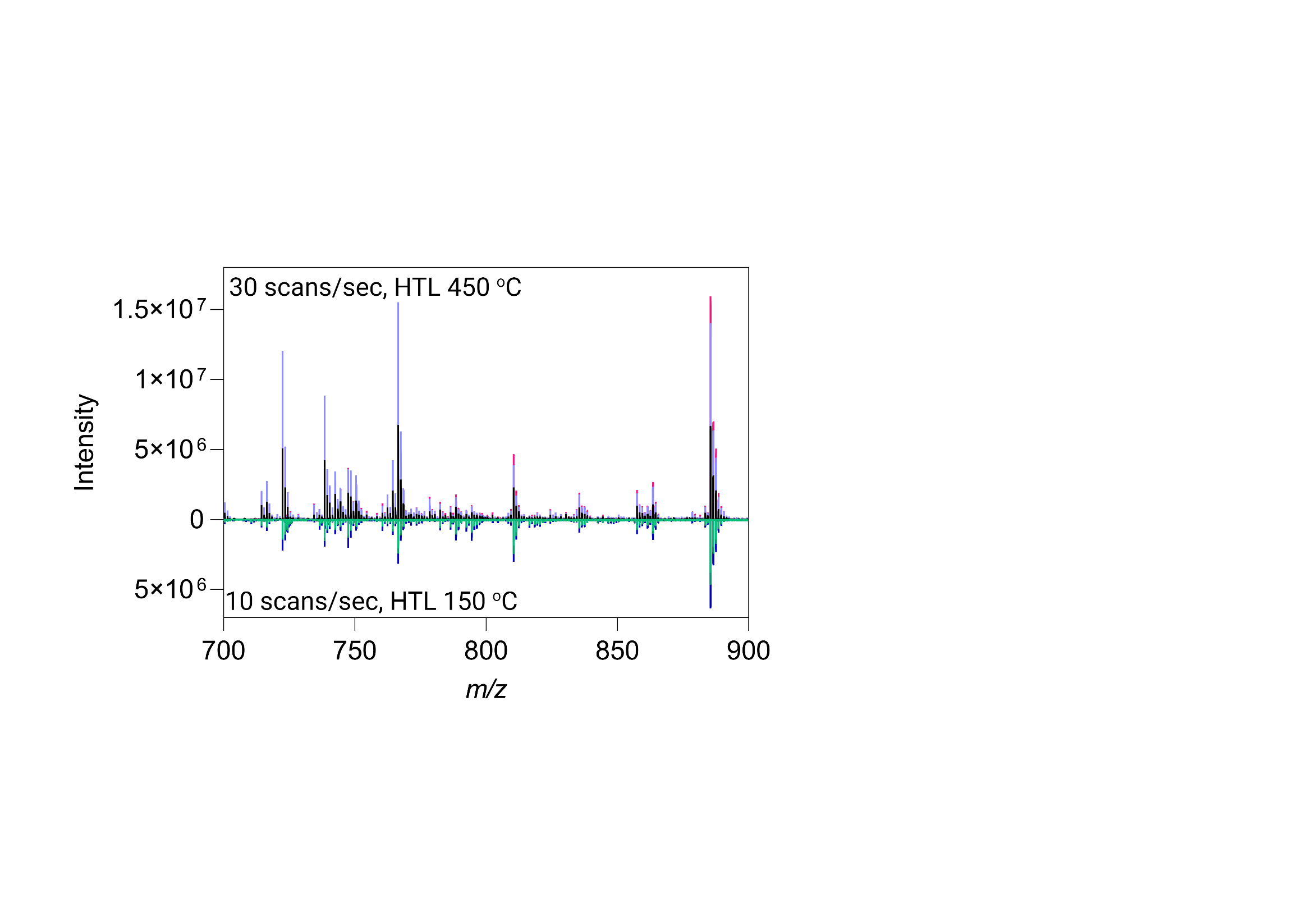


**Supplementary Fig. S2** **DESI-MS spectra of kidney tissue obtained with heated transfer line (HTL) set to 450 ^o^C and scan speed of 30 scans/sec (pink, purple and black spectra), and at 150 ^o^C and 10 scans/sec (blue, light and dark green spectra)**. We used data from the previous experiment for the transfer line temperature at 150 ^o^C for this comparison. Same size ROI were drawn on the same region of all 6 sections. As sections are not consecutive but are from the same tissue, we compared the spectra of sections scanned at 10 scans/sec at 450 ^o^C for the transfer line experiment with the spectra of the replicates of the 10 scans/sec scan speed experiments (data not shown). As intensities were consistent between both experiments, we concluded that a comparison between 150 ^o^C at 10 scans/sec and 450 ^o^C at 30 scans/sec was appropriate


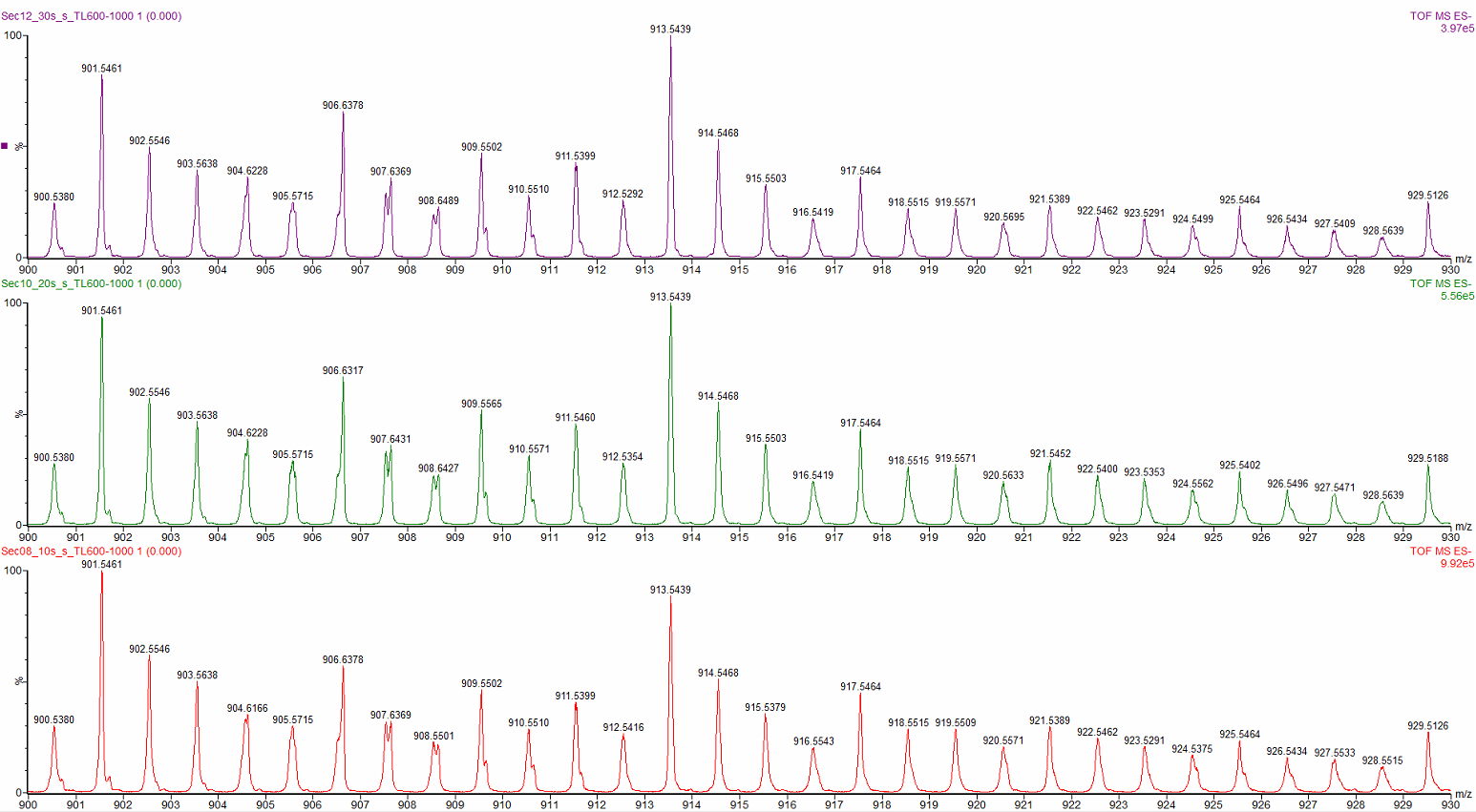


**Supplementary Fig. S3** **Illustration of peak shape consistency for low-intensity peaks at different scan speeds in the range m/z 900-930**. Red spectra show data acquired at 10 scans/sec, green spectra at 20 scans/sec and purple spectra at 30 scans/sec


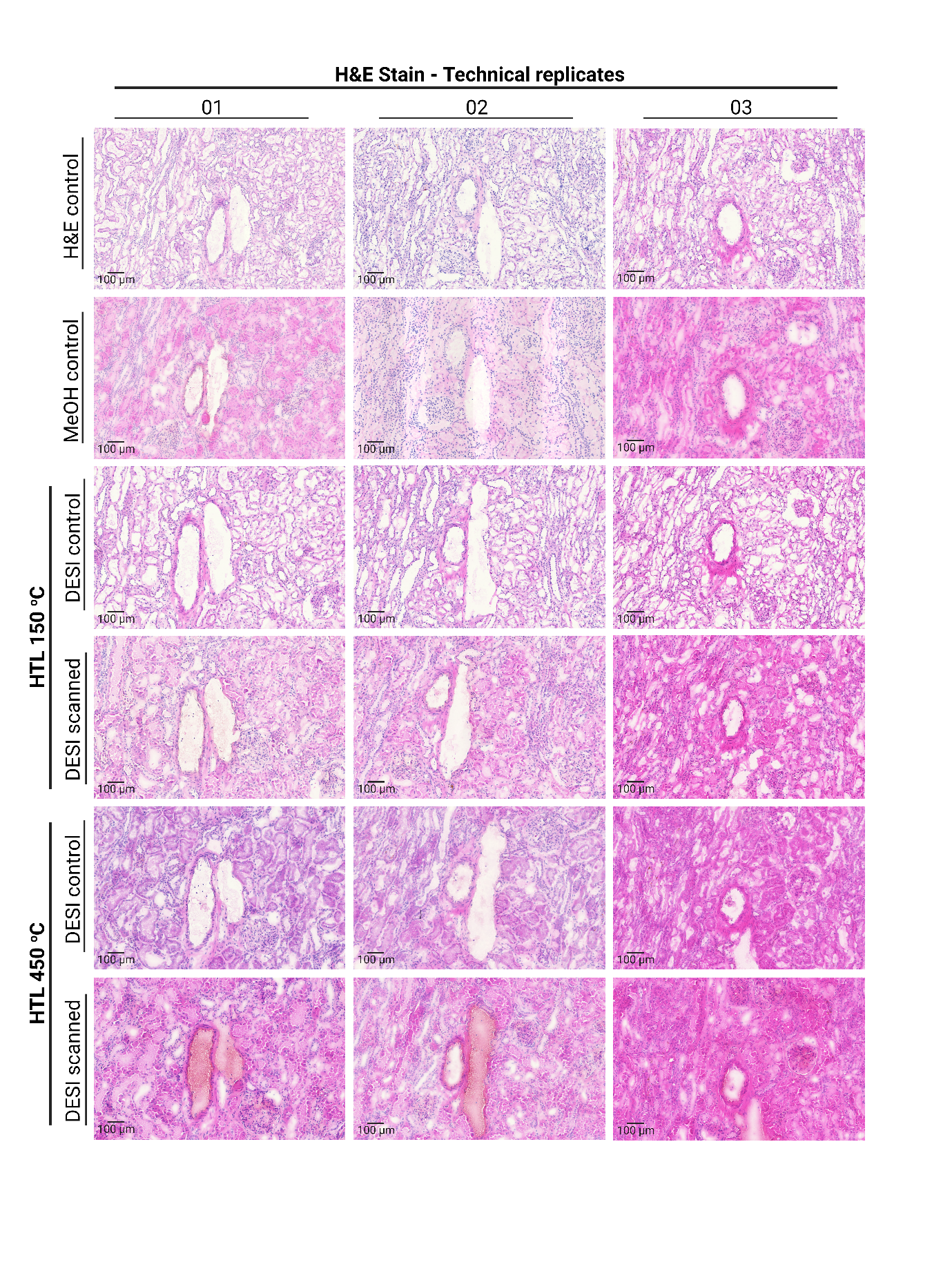


**Supplementary Fig. S4** **H&E histopathology assessment of snap-frozen kidney tissue sections submitted to DESI-MSI analysis.** DESI-scanned sections were analysed under the same DESI and MS conditions varying only the heated transfer line (HTL) temperature. DESI control sections were kept inside the enclosed DESI source whilst scanning DESI-scanned sections to evaluate the effect of the environment temperature and solvent on tissue histology


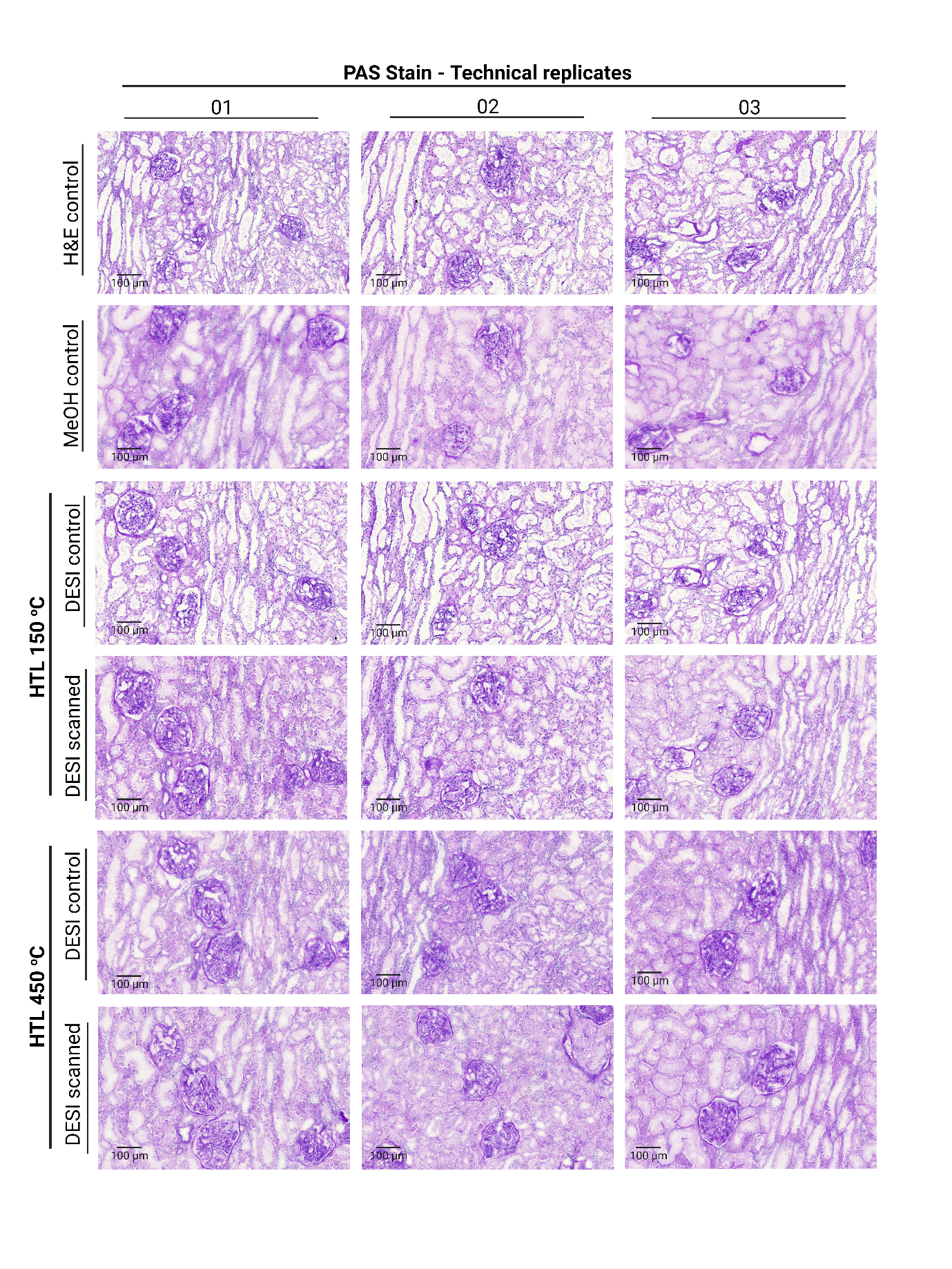


**Supplementary Fig. S5**  **PAS histopathology assessment of snap-frozen kidney tissue post-DESI-MSI and H&E staining.** DESI-scanned sections were analysed under the same DESI and MS conditions varying only the heated transfer line (HTL) temperature. DESI control sections were kept inside the enclosed DESI source whilst scanning DESI-scanned sections to evaluate the effect of the environment temperature and solvent on tissue histology


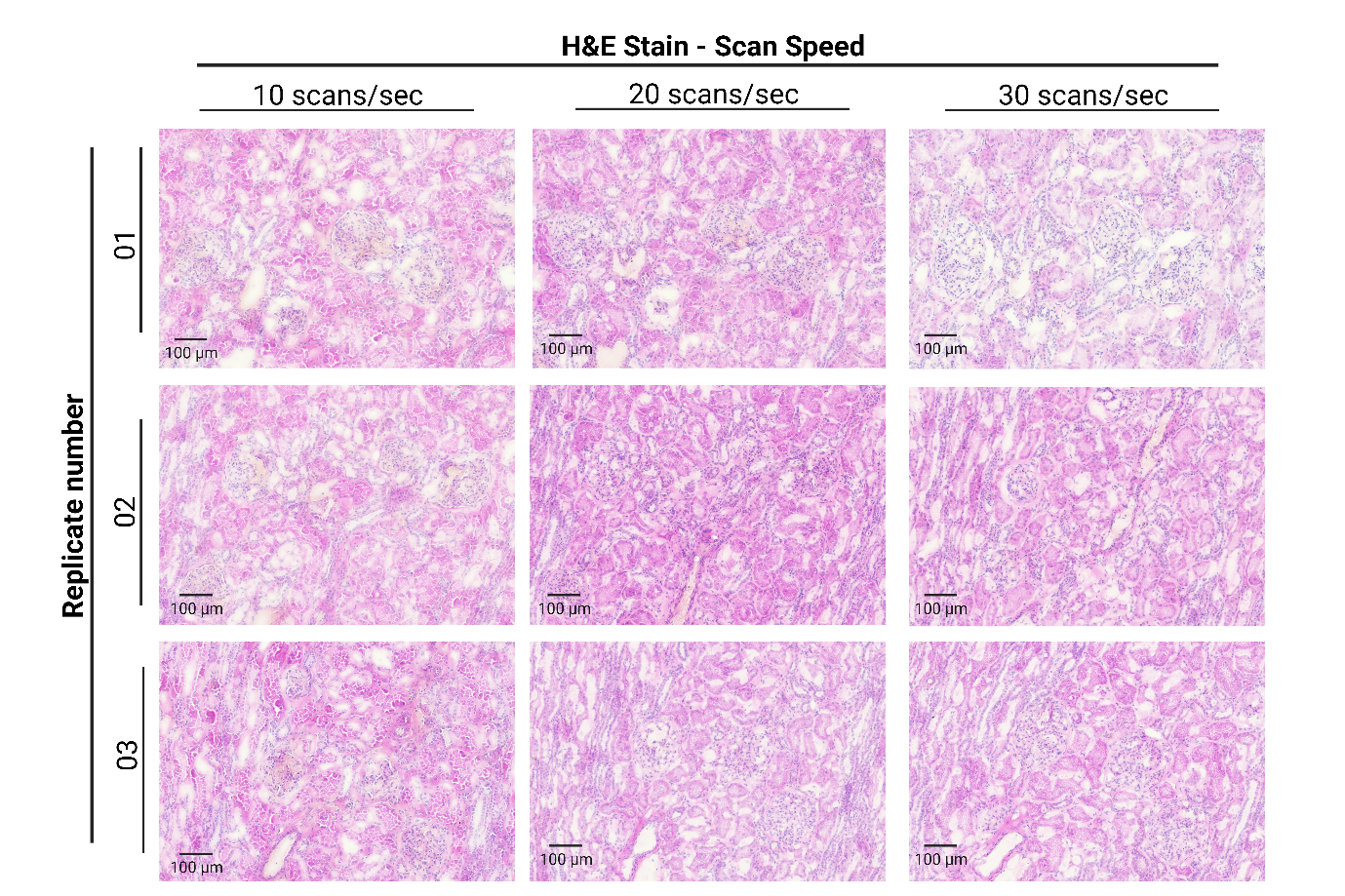


**Supplementary Fig. S6** **H&E histopathology assessment of the effect of DESI-MSI scan speeds on snap-frozen kidney tissue sections.** DESI-scanned sections were analysed under the same DESI and MS conditions with the heated transfer line (HTL) at 450 ^o^C varying only the acquisition rate from 10, 20 and 30 scans/sec


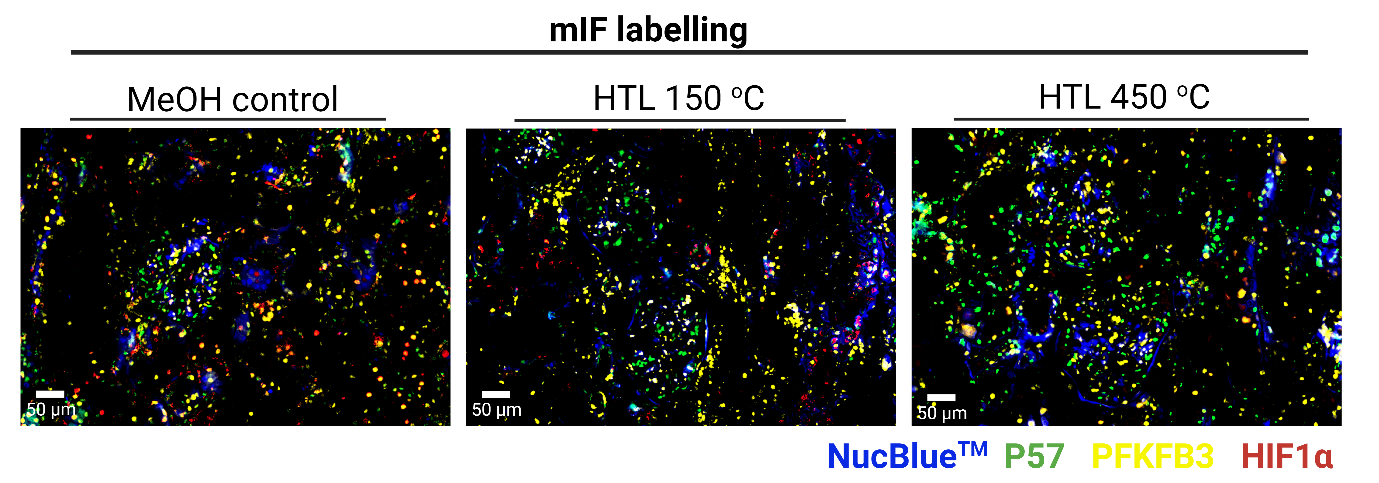


**Supplementary Fig. S7** **mIF labelling of kidney sections post-DESI, H&E and PAS staining.** Composite mIF image of podocytes (P57^+^, green labelling), PFKFB3^+^ (glycolysis, yellow labelling), HIF1α^+^ (hypoxia, red labelling) cells and nuclear marker (NucBlue™)


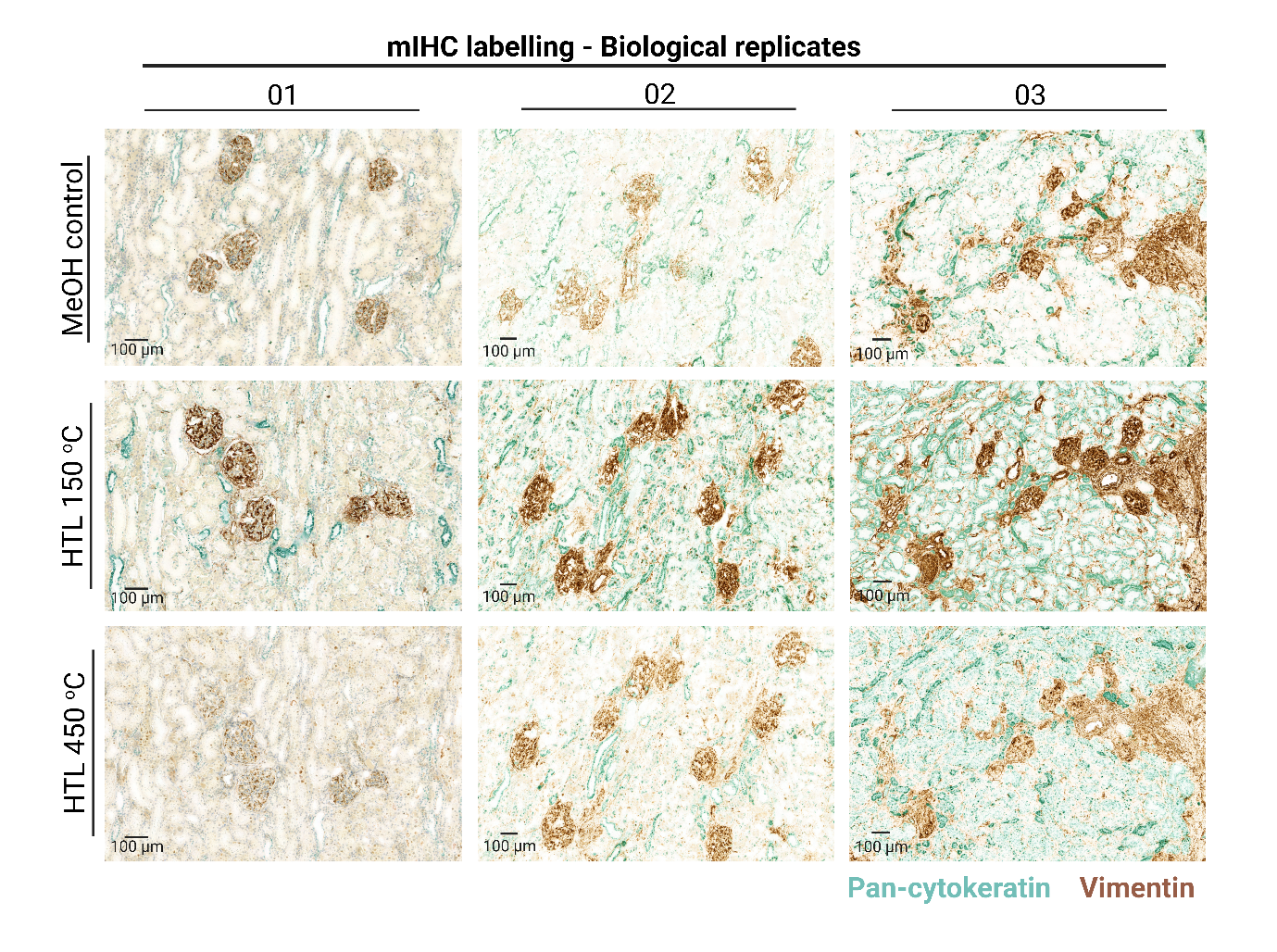


**Supplementary Fig. S8** **mIHC labelling of pan-cytokeratin and vimentin on kidney tissues post-DESI-MSI analysis, H&E and or/PAS staining and mIF labelling.** mIHC labelling on sample 01 was performed after DESI-MSI analysis, H&E and PAS staining and mIF labelling (technical replicates from sample 01 not shown). mIHC on samples 02 and 03 was performed post-DESI-MSI analysis, H&E staining and mIF labelling


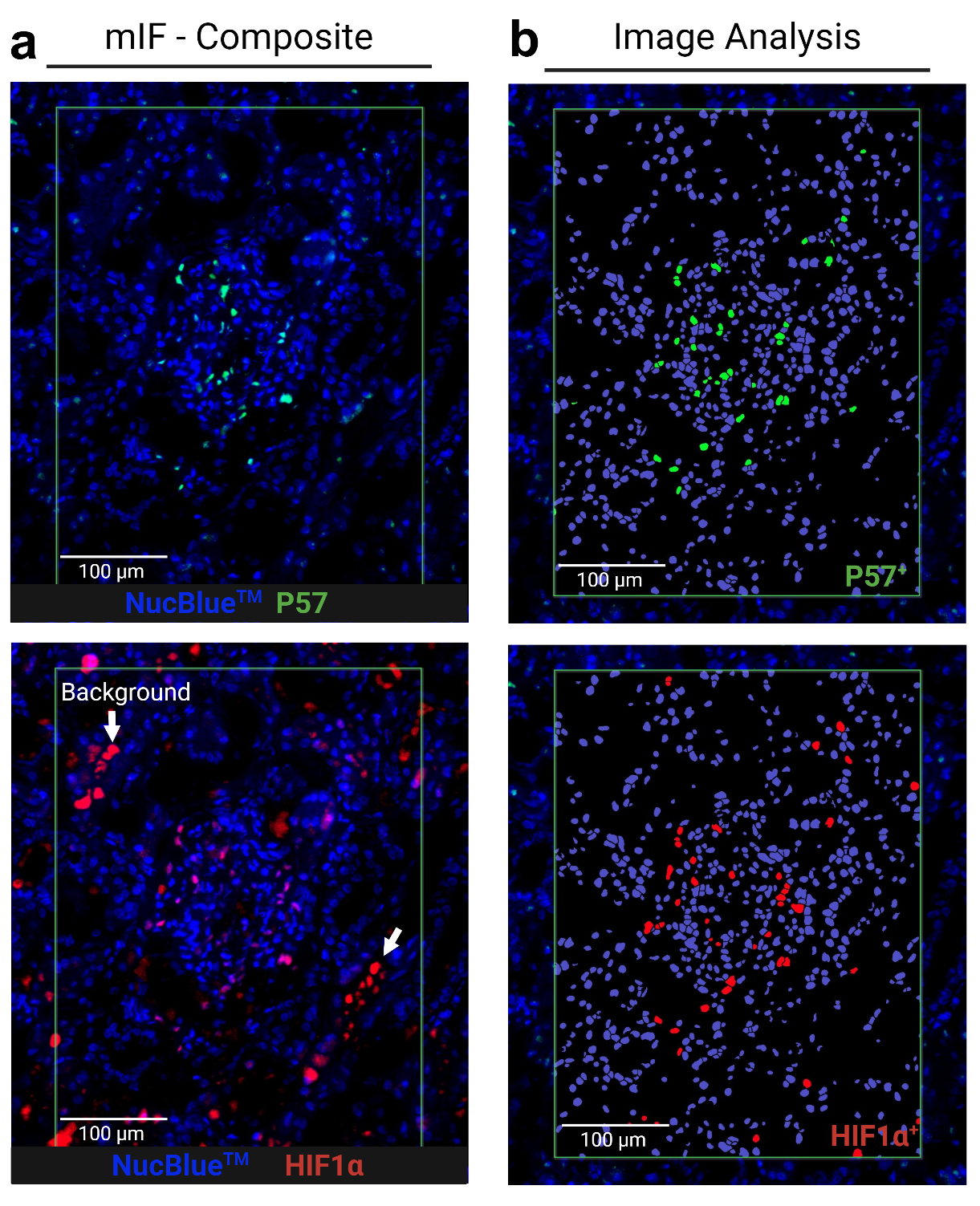


**Supplementary Fig. S9** **Image analysis for identification of p57^+^ and HIF1α^+^ cells.** **(a)** mIF images of nuclear marker (NucBlue™), podocytes (P57^+^, green labelling) and HIF1α^+^ cells (red labelling) of the same region of a tissue analysed by DESI-MSI with the HTL at 450 ^o^C at 10 scans/sec. **(b)** AI-enabled nuclear segmentation and identification of cells positive for P57 and HIF1α


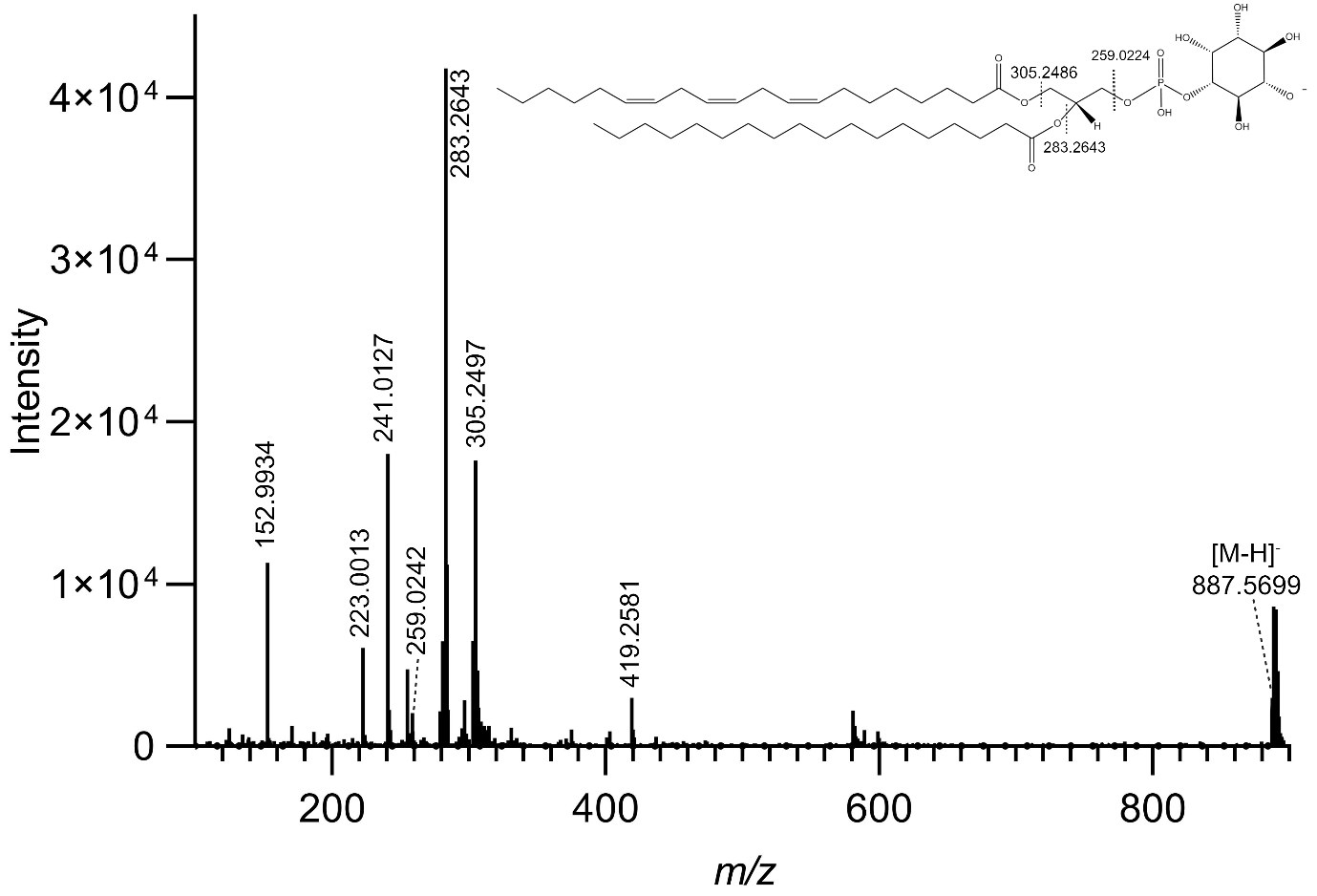


**Supplementary Fig. S10** **DESI-MS/MS spectrum of PI 38:3 (m/z 887.5620).** The lipid identification using DESI-MS/MS was confirmed as phosphatidylinositol PI(18:0_20:3), where the m/z fragment 259.0242 corresponds to the inositol phosphate ion, m/z 305.2497 to the sn1 RCOO^-^ ion [FA 20:3-H]-, m/z 283.2643 to the sn2 RCOO^-^ ion [FA 18:0-H]-, m/z 241.0127 and 223.0013 correspond to the inositol phosphate ion with one and two H_2_O molecules, respectively, m/z 152.9934 to glycerol-3-phosphate ion with loss of H_2_O and m/z 419.2581 to the neutral loss of sn1 RCOO^-^ group and inositol.


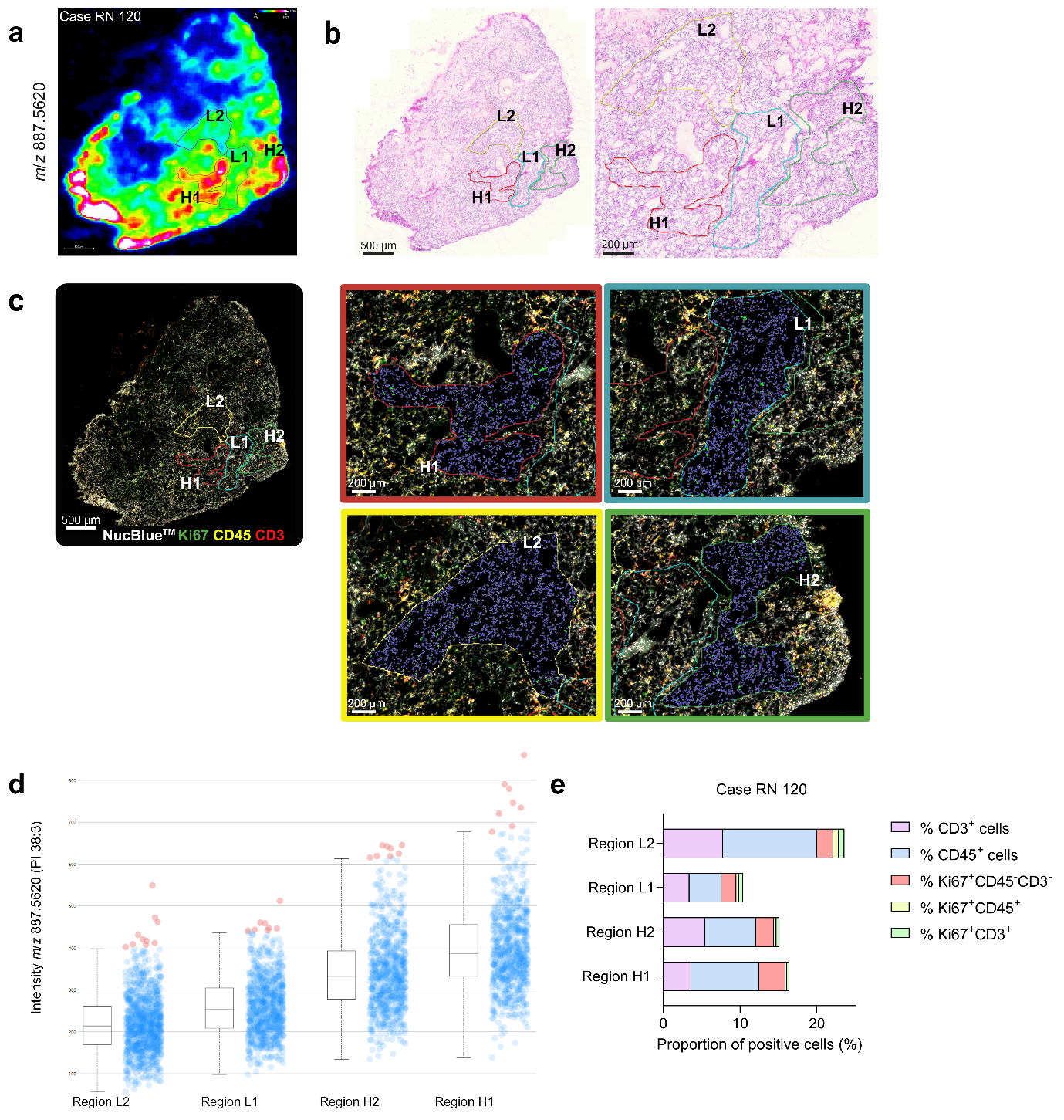


**Supplementary Fig. S11** **Multimodal imaging to evaluate the correlation between PI 38:3 intensity with Ki67^+^ CD45^-^ CD3^-^ cells in ccRCC case RN 120.** (a) Ion image of *m/z* 887.5620 (PI 38:3) of case RN 120 and selected regions of interest (ROI) considered of high (H1 and H2) and low (L1 and L2) ion intensity. (b) H&E-stained section post DESI-MSI highlighting ROIs of high (H1 and H2) and low (L1 and L2) PI 38:3. (c) composite image of mIF labelling, post-DESI and H&E staining, of Ki67^+^ (green), CD45^+^ (yellow) and CD3^+^ (red) cells and image analysis results of the ROIs of high and low PI 38:3 highlighting in green Ki67^+^ CD45^-^ CD3^-^ cells. (d) *m/z* 887.5620 ion intensity of the analysed ROIs. (e) Percentage of positive cells for each analysed phenotype amongst the total number of cells in the ROIs


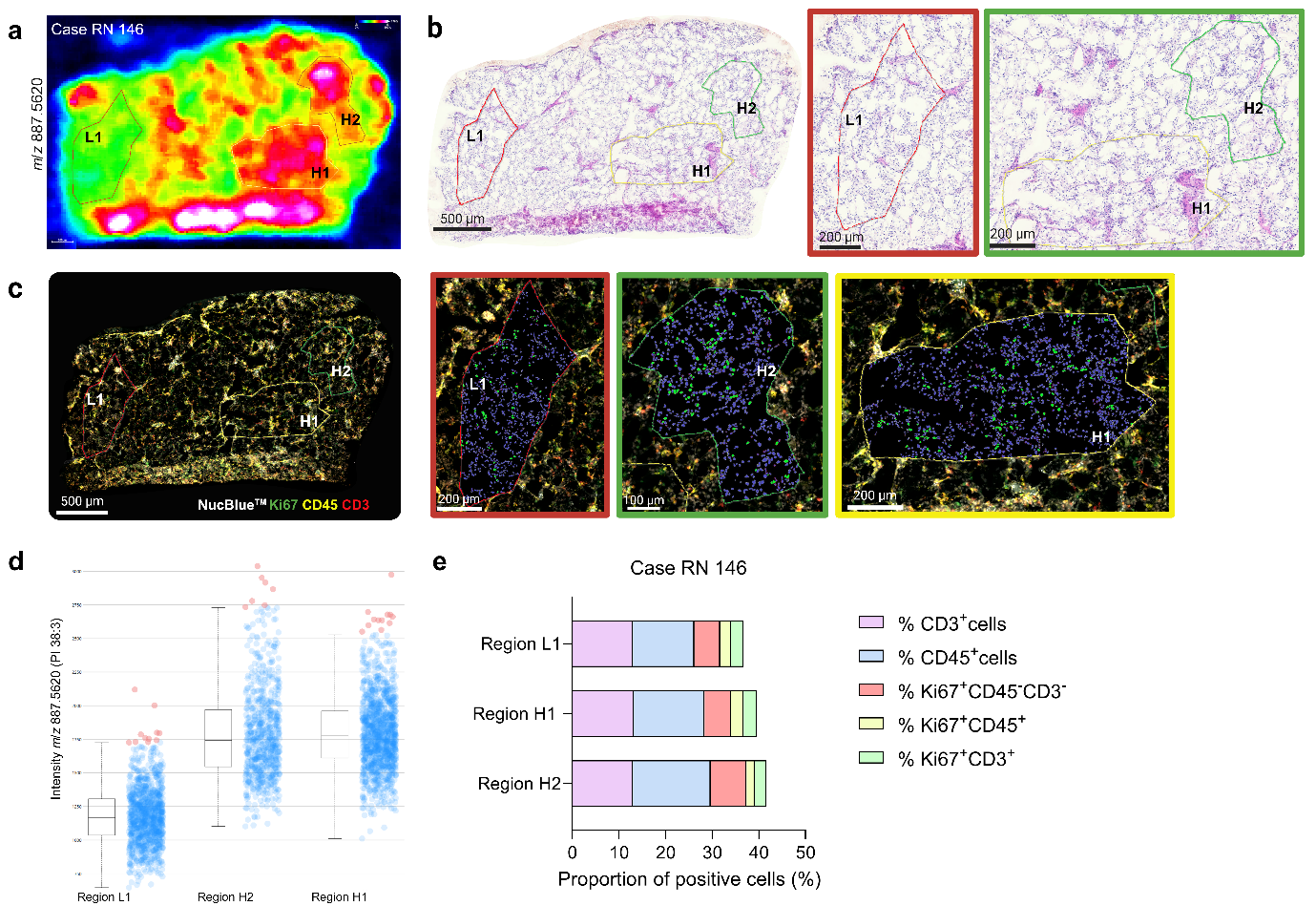


**Supplementary Fig. S12** **Multimodal imaging to evaluate the correlation between PI 38:3 intensity to Ki67^+^ CD45^-^ CD3^-^ cells in ccRCC case RN 146.** (a) Ion image of *m/z* 887.5620 (PI 38:3) of case RN 146 and selected regions of interest (ROI) of high (H1 and H2) and low (L1) ion intensity. (b) H&E-stained section post DESI-MSI highlighting ROIs of high (H1 and H2) and low (L1) PI 38:3. (c) composite image of mIF labelling, post-DESI and H&E staining, of Ki67^+^ (green), CD45^+^ (yellow) and CD3^+^ (red) cells and image analysis results of the ROIs of high and low PI 38:3 highlighting in green Ki67^+^ CD45^-^ CD3^-^ cells. (d) *m/z* 887.5620 ion intensity of the analysed ROIs. (e) Percentage of positive cells for each analysed phenotype amongst the total number of cells in the ROIs
